# Distribution pattern and driving factors of mite communities in karst cave ecosystems

**DOI:** 10.1002/ece3.11527

**Published:** 2024-08-07

**Authors:** Yifan Fei, Zheng Shi, Yuanyuan Zhou, Qiang Wei, Ying Liu, Yan Shen, Hu Chen

**Affiliations:** ^1^ School of Karst Science Guizhou Normal University Guiyang China

**Keywords:** biodiversity, cave ecosystems, cave mites, ecological linkages, environmental factors

## Abstract

Mites are among the most abundant invertebrates in subsurface ecosystems, and their community assemblages and distributions are often significantly influenced by the diversity of habitat resources. The cave ecosystem encompasses drastic changes in nonbiological factors, such as changes in lighting conditions from bright to extraordinarily dark and habitat gradients of surface plant resources from abundant to scarce or even disappearing, providing an ideal unique environment for evaluating the assembly mechanism of soil animal communities. Nevertheless, there still needs to be a sufficient understanding of the biodiversity patterns and drivers of mite communities across environmental gradients in karst caves. We conducted a comprehensive survey on the composition and diversity of soil mites in three photometric zones (dark, twilight, and light) of a typical karst cave and its adjoining extractive environments (forest scrub and farmland). Our research aimed to investigate the ecological relationships of mite communities between above‐ and below‐ground habitats and the effects of abiotic factors on mite communities. We collected 49 families, 86 genera, and 1284 mites. In the external cave environment, we captured 1052 mites from 72 genera and 45 families; in the internal cave environment, we captured 232 mites from 46 genera and 29 families. The abundance, richness of genera, and diversity parameters of the mite community decreased from the cave entrance to the cave interior with decreasing light intensity. Oribatid mites dominated the mite community. *Protoribates* and *Scheloribates* were the dominant genera, along with *Tectocepheus* and 11 other genera, which primarily distinguished the mite communities among different habitats. Forty endemic taxa were found in the external cave environment, compared to 14 endemic taxa in the internal cave environment. The mite community showed a strong preference for the cave ecosystem habitat. Temperature, humidity, and soil nitrogen content significantly influenced the distribution pattern of mite communities (VIP > 0.8, *p* < 0.05).

## Graphical abstract

Mites are abundant invertebrates in underground ecosystems, influenced by resource and environmental diversity, but the diversity of cave mites remains unclear. Through a survey of soil mites in three typical areas of light intensity in caves and their adjacent environments outside the caves, we found that although there are significant differences in mite community diversity and species composition under different environmental conditions, there is still a specific correlation between the mite communities inside and outside the caves. In addition, there remains a stable zone between the two, which is a transitional zone for the survival of mite communities. Mite communities have a high degree of selectivity toward cave environments. The adapted environmental conditions have a positive effect on the mite community. These findings contribute to understanding mites' global ecological distribution patterns and provide a scientific basis for protecting cave biodiversity and ecosystem health.

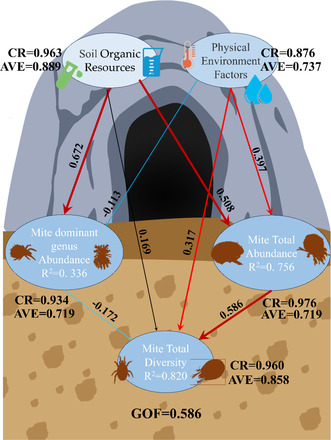

## 1 INTRODUCTION

Resource and environmental heterogeneity are significant drivers of biodiversity (Barreto & Lindo, 2018; Eisenhauer et al., 2010), where the diversity of below‐ground habitats is recognized to be closely related to above‐ground plants. Thus, it is often assumed that plant diversity contributes to the heterogeneity of food sources and microhabitats, increasing soil fauna diversity (Bluhm et al., 2019). However, compared to the extensive vegetation cover of surface habitats, caves form through chemical reactions between circulating groundwater and surrounding rocks, resulting in isolated and strongly zoned environments (Pu et al., 2024; Romero, 2009). These environments are extreme, relatively closed, and stable, with limited resources and unique physical conditions (Martin‐Pozas et al., 2024), rendering them distinct habitats in ecosystem research (Darwin, 1859; Mammola et al., 2020). Cave ecosystems have a simple nutritional structure, with few individual species (Romero, 2009). Despite this, they harbor a vast biodiversity, estimated to exceed 50,000 species globally (Culver & Pipan, 2009), turning them into natural laboratories for ecological, evolutionary, and biomedical research (Recknagel & Trontelj, 2021). Cave environments are characterized by minimal diurnal and annual temperature and humidity fluctuations, dim lighting, nutrient deficiencies, and limited plant presence, typically concentrated in illuminated zones (Monro et al., 2018; Romero, 2009; Souza‐Silva et al., 2021). These features create pronounced resource‐environment heterogeneity within caves. With decreasing light, resource scarcity further divides caves into twilight and dark zones, making them ideal for studying biological community adaptation and ecological relationships (Wilkens et al., 2000). Presently, cave biology primarily focuses on describing and interpreting species diversity, a vital aim of this study (Culver & Pipan, 2009). Environmental differences between internal and external cave environments and photic zones have resulted in varied animal adaptive characteristics. Schiner (1854) proposed a classification system based on the degree of cave environment dependency, dividing cave animals into three groups: troglobites, troglophiles, and trogloxenes. There is a close interconnection among these groups, with a continuous transition from epigean animals to epigean fauna, with trogloxenes and troglophiles in between (Liu, 2021). However, little is known about the ecological relationship between troglofauna and epigean fauna (Skuba et al., 2013). Previous research indicates that resource heterogeneity can result in variations in species composition and diversity among faunal communities (Souza‐Silva et al., 2021; Zhou et al., 2022). Significant environmental disparities between cave interiors and exteriors can influence faunal community distribution patterns (Gong et al., 2023; Yang et al., 2021). Hence, further investigation into how environmental factors shape faunal community distribution patterns is crucial for comprehending their ecological roles in cave ecosystems. However, contemporary studies on cave fauna taxa predominantly concentrate on cave fishes (Torres‐Dowdall et al., 2018), bats (Liu, 2021), etc., while invertebrate arthropods, such as soil mites, which serve as excellent ecological indicator organisms, have yet to be systematically studied.

Soil mites, belonging to the phylum Arthropoda and the order Acari formes, are one of the most abundant and diverse groups of soil animals and one of the main research hotspots in soil animal ecology (Walter & Proctor, 2010). Soil mites are a vulnerable community of organisms in the soil environment and are sensitive to changes in the soil environment (Meehan et al., 2019). In surface habitat studies, soil mites have been widely used to indicate gradient changes in forest ecosystems (Zhou et al., 2022), disturbances from agricultural activities (Wei et al., 2022), ecological restoration of degraded ecosystems (Liu et al., 2022), ecological succession, and pest control, among other fields (Chen et al., 2023). They are considered one of the most critical indicators for environmental monitoring (Walter & Proctor, 2010). In contrast, research on subterranean habitats has been largely unsystematic, studies on cave mites were carried out relatively late, with the description of Belba lengersdorfi in 1932 by Willmann in a stalactite cave being the first record of an armored mite in a cave (Willmann, 1932). Since then, studies on mites have been carried out in caves in Europe, Asia, North America, and Brazil but have mainly focused on the mite fauna and the publication of new species (Bruckner, 1995; Nakamura et al., 2010; Wauthy & Ducarme, 2006). By 2021, about 1000 species of ticks and mites are known from caves (Liu, 2021), while studies on the ecological relationships of mites are still scarce, which may be related to the difficulty of sampling caves and the high heterogeneity of the environment. There are only a few mite studies in the literature on cave habitats in China; Yang (Yang et al., 2021) collected four cave mites in Guizhou, China, and described 20 families of armored mites, Gong (Gong et al., 2023) compared cave moss mites in caves with surface habitat moss mites, and Fu (Fu et al., 2023) documented the first occurrence of genera in caves in Yunnan, China and new species, and published the first taxonomical study of cave Acari Oribatida in southwest China. Other than that, there needs to be more information on collecting cave mites and describing taxa.

China's 1.9 million square kilometers of karst area is one of the world's most threatened biodiversity hotspots (Duan et al., 2021); in the fragile ecological background of karst, unreasonable human activities have caused severe degradation of karst ecosystems (Brandt et al., 2018), especially the karst geomorphic environment is a dichotomous structure consisting of two major systems, surface and subsurface, and the cyclic interaction of the two is complex and rapid, which also provides a pathway for the flow of pollutants, resulting in karst environments that are easy to pollute but difficult to manage (Yang, 1990). In our investigation, some caves have been disturbed by human activities, the most prominent of which is garbage dumping. Although a small portion of the Southern China Karst was listed as a UNESCO World Heritage Property in 2007 (UNESCO, 2007) and 2014 (UNESCO, 2014), most of China's caves are not effectively protected and face high threats to cave ecosystems from damage caused by karstic action (Yuan‐Hai & De‐Hao, 2012), human agricultural activities (Neill et al., 2004), rubbish dumping (Shu et al., 2013), tourism (Monro et al., 2018), and other impacts (Duan et al., 2021). Hence, it is of great significance for the ecological environment and biodiversity conservation in the karst region to explore the ecological relationship between soil mites in their subterranean and surface habitats by researching them (Moseley, 2009b). However, the research has not been carried out systematically, even worldwide (Barczyk & Madej, 2015; Skuba et al., 2013).

In the research, we selected three typical karst caves. We investigated soil mites in different photometric zones (light, twilight, and dark zones) inside the caves and in the external space immediately adjacent to the caves (scrub, cornfield, and cabbage field). We explored the variation and composition of soil mite communities, focusing on adapting mite community diversity to cave environments and proposing the conservation of cave ecosystems. As a result, we formulated three hypotheses: (1) The soil mite communities' diversity and composition differ significantly under different environmental conditions. (2) The soil mite communities in the internal environment of caves and those in the external environment of caves are closely linked in ecological relationships. (3) In cave ecosystems, the diversity and species composition of soil mite communities were significantly affected by the content of organic resources or the physical environment.

## 2 MATERIALS AND METHODS

### 2.1 Overview of the study area

Southern China Karst is the world's most typical and complex karst development, with the most significant number of landscapes and ecological types (Xiong et al., 2008). Guizhou Province, as a typical distribution area of karst in southern China, the karst landscape covers about 73.8% of the province's land area (Zhou, 2004), which also provides good geological conditions for the development of caves. It is estimated that there are about 1.8 million caves now distributed around the world (Monro et al., 2018), while the number of existing recorded caves in Guizhou province has reached more than 6000. Caves are distributed in basically all the areas of the province (Zhang et al., 2016).

**FIGURE 1:**
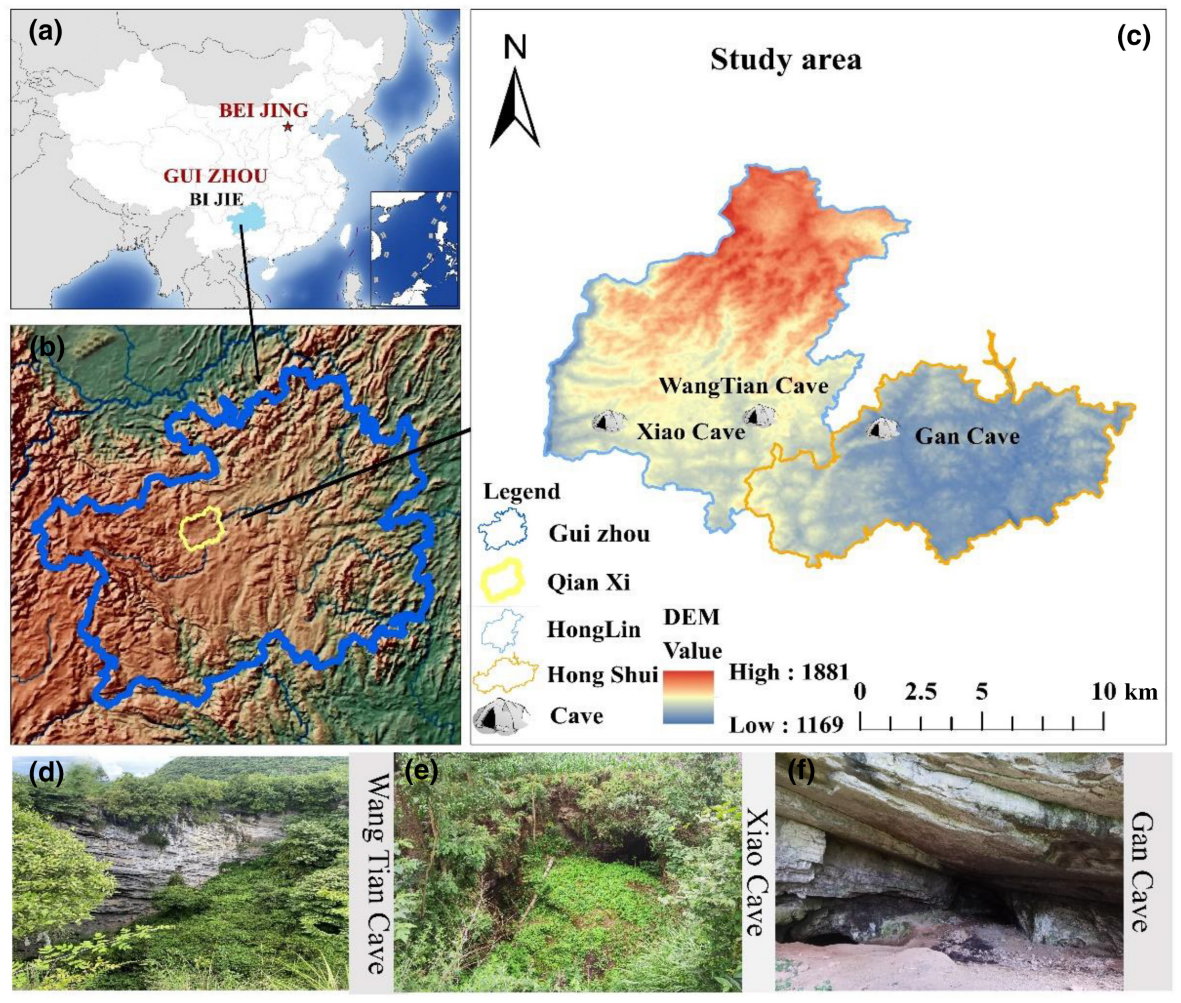
Overview of the study area. *Note*: Geographic location of Guizhou Province (a), geographic location of Qianxi City (b), geographic location of the research caves (c), Wangtian Cave (d), Xiao Cave (e), and Gan Cave (f). The external environments of the three research caves are shown at the top of the locality map.

The sampling locations are in Honglin Yi and Miao Ethnic Township (later referred to as Honglin Township) and Hongshui Town (later referred to as Hongshui Town) in Qianxi City, Guizhou Province, China (Figure 1). Honglin Township is located in the western part of Qianxi City, covering an area of 103.22 square kilometers. The terrain in the region is dominated by plateaus, mountains, and hills, belonging to a karst‐fragile environment. The terrain is higher in the northwestern part and flat in the central part. The average annual temperature is 13°C, with an average annual precipitation of 1010 mm and an average frost‐free period of 270 days per year. Hongshui Township is situated in the northwestern part of Qianxi City, covering an area of 70.44 square kilometers. Most of the territory consists of hills and basin lands, with a terrain higher in the west and lower in the east. The altitude across the township ranges between 1100 and 1600 m, and the average annual temperature is 14°C. The annual precipitation averages 1100 mm, and the average frost‐free period is 280 days. The carbonate rocks in the area are widely spread, and mostly exposed, with more than 85% of the distribution area, and most of them are pure carbonate rocks.

### 2.2 Sample selection and settings

In June and July 2023, we selected three karst caves in the area: Wangtian Cave, Gan Cave, and Xiao Cave. At the same time, we used the DLX‐YQ2901B GPS instrument to record the coordinates and elevation, of each cave, measured with a DLX‐D laser rangefinder the orientation of the cave entrance, and the degree of disturbance inside and outside the cave. We investigated the distribution of vegetation inside and outside the cave (Appendix S1: Table A1). The internal environment of the caves was divided into a light zone (light >10 lx), a twilight zone (light 0.1–10 lx), and a dark zone (light <0.1 lx) based on light intensity (Li et al., 2001). This research used light intensity as the gradient for sampling the cave interior environment. The reason for such a setting is that there tends to be some small amount of vegetation distribution in the light zone. At the same time, some scholars believe that the cave's light zone is a transition zone connecting the above‐ground and below‐ground areas. The twilight zone and the dark zone are viewed as the deeper space of the cave, which is the typical subterranean habitat study area (Moseley, 2009b; Romero, 2009; Skuba et al., 2013). Outside the caves, we collected adjacent farmland and shrubs as control environments. We collected 13 plots, 117 sampling points, and 234 soil mite samples from three caves during the study. Among them, there were 30 sampling points outside the caves, with 18 sampling points in the light zone, 18 in the twilight zone, and 51 in the dark zone.

**FIGURE 2:**
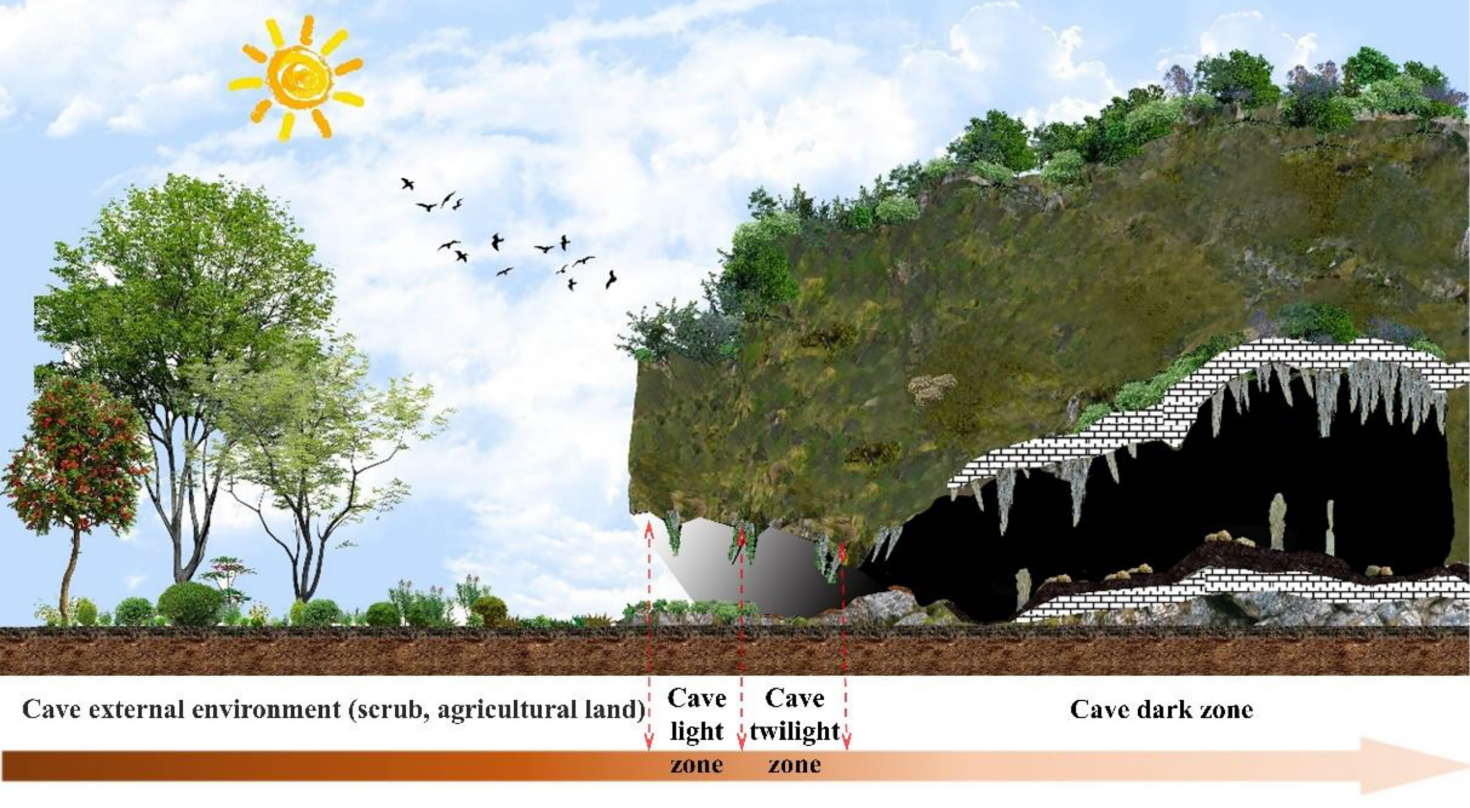
Distribution map of cave sample site collections (see Appendix S1 for habitat images of sample sites).

In this research, we designed the sampling for the actual situation of the internal environment of each cave separately (Table 1, Figure 2). Wangtian Cave is a sinkhole‐type cave in Xinping Village, Honglin Township (105°53′38″ E, 27°6′25″ N). There is a certain amount of domestic rubbish at the mouth of the cave in the karst sinkholes the slope leading to the bottom of the pit is littered with domestic waste, whereas the caves at the bottom of the karst sinkholes are largely undisturbed by human activity. The average height difference from the top of the sinkhole to the bottom of the cave is about 100 m, and there is a large amount of vegetation distributed within the sinkhole (Figure 1d, Appendix S1) and at the bottom of the sinkhole, there are two openings, which are divided into the East Side Cave and West Side Cave, and there are mosses, lichens, etc., distributed in the opening of the East Side Cave. There are many scattered rocks at the cave entrance, and the twilight zone is mainly composed of sediment accumulation and sediment from underground rivers with high humidity. A part of the dark zone is the rimstone dam, and there is a slope mixed with rocks and soil on the side of the edge stone dam, with a slope of 70–35° and a height difference of about 25 m. The top of the slope is relatively flat, and after passing through a 10‐m passage, there is a flat area about 120 m long and 6–25 m wide. We designed three sample strips in the dark zone, and each sample strip was set up with a sampling point at an interval of 10 m for a total of 33 sampling points. In the twilight zone, 12 sampling points were randomly set up, each at an interval of more than 1 m. Six sampling points were set up for the light zone, the corn field outside the cave, the scrub outside the cave, and the plants outside the cave are shown in Table A1 in Appendix S1.

**TABLE 1:** Types of sample sites corresponding to the three caves and their corresponding sample site numbers.

Cave	Wangtian cave		Gan cave		Xiao cave	
Samples	Scrub	A1	Arboreal	B1	Cabbage field	C1
	Corn field	A2	Herbaceous	B2	Light	C2
	Light	A3	Light	B3	Dark	C3
	Twilight	A4	Twilight	B4		
	Dark	A5	Dark	B5		

*Note*: All numbering in the following figure represents consistency.

Gan Cave, located in the town of Hong Shui (105°56′31″ E, 27°5′57″ N), the cave entrance faces south‐west and is at the top of a hillside. The cave has a history of saltpeter making in the last century; the presence of human interference is largely unnoticed at this stage, and trees, scrub, and herbaceous plants are distributed in the immediate vicinity of the cave's exterior. The entrance of the cave has a light zone with bats roosting. After a section of gentle slope (height difference of about 3 m, slope of 20–35°) connecting the deep space, the topography of the twilight zone of the cave is flat, there are soils and travertine distributions, immediately adjacent to the deep part of the twilight zone there is a curved channel with a height of 1.5–1.8 m and a width of 3–8 m, considering its close proximity to the entrance of the cave, a sample site for the dark zone sampling is set up at this place. The deep space of the cave is more complex, distributed with rimstone dam, stalactite, subterranean stream, and other kinds of topographic units in the dark zone at the bottom of the cave, for the soil and stone mixed area, containing a small amount of travertine, we also set up a sample plot of the dark zone, the dark zone is set up in a total of 12 sampling points. Six sampling points were set up for the twilight zone, the lighted zone at the cave entrance, the herbaceous zone outside the cave, and the arboreal zone outside the cave.

Xiao Cave, located in Hongwafang Village (105°50′25″ E, 27°6′12″ N), Honglin Township, is a sunken cave next to the road with two entrances; the right‐side cave has only six sampling points because a collapsed stalactite covers the vast majority of its internal space, and the sampling area of the dark zone is small. The left side cave has a karst window present, there is a severe anthropogenic disturbance, a large amount of rubbish was discarded in the interior of the cave through the karst window, and the left side cave was a cave where local nitrate refining was carried out in the last century, and there is no suitable collection site, so no sample area was set up. There is no soil distribution in the twilight zone of this cave, and six sampling sites were set up in the light zone immediately adjacent to the cave entrance in the cabbage field.

Soil samples from the 0–5 and 5–10 cm layers were collected at each sampling site using a ring knife (a cylindrical sampler with a diameter of 10 cm and a height of 5 cm) with a volume of 500 cm^3^. The samples were placed in well‐ventilated cotton bags to prevent soil mites from escaping and were brought back to the laboratory for mite extraction.

In order to compare the differences in soil properties inside and outside the caves and with mite diversity near each sampling site, we measured air temperature (AT), air humidity (AH), and soil temperature (ST). We collected a sample of about 500 g of soil physicochemical properties to return to the laboratory for processing.

### 2.3 Laboratory processing

A modified Berles‐Tullgren baking funnel was used to isolate soil mites in the soil fauna isolation laboratory. Based on the pre‐experiment of the pre‐sampling, we concluded that the temperature of mite extraction inside and outside the burrow should be controlled separately. For mite extraction outside the burrow and those with a light band, we controlled the temperature in the baking cabinet to 38°C. We baked the mites continuously for at least 48 h (until the soil samples were completely dry). The pre‐experiment experiment found that the degree of ossification of mites in the dark and twilight zones was low and easy to break. At the same time, considering the low temperature and high humidity in the deep space of the cave itself, we controlled the temperature at 30°C when extracting mites in the dark and twilight zones to prevent their death. We baked them continuously for 72 h (until the soil samples were completely dry).

The extracted mites were stored in a 75% ethanol solution. Soil mites were isolated under a stereomicroscope (Olympus SZX2‐FOF). For mites outside the cave, darker‐colored mites were stored in a 95% lactic acid solution for 15 days before identification. However, for mites inside the cave, considering that lactic acid solution can reduce the degree of ossification of mite samples and make them easily fragmented, samples inside the cave were kept in a 75% ethanol solution.

The moss mites samples were made into temporary pieces. The sources used for identification were *A Manual of Acarology*, *Pictorial Keys to Soil Animals of China*, S*oil Gamasid Mites in Northeast China* and *Acarology* (Krantz & Walter, 2009; Li & Li, 1989; Yin et al., 2013; Ying, 1998). All moss mites were identified to taxonomic units at the genus level, except for juvenile moss mites and incomplete specimens, which were difficult to identify reliably. The moss mites specimens were kept in the laboratory of the School of Karst Science, Guizhou Normal University, China.

A portion of the collected soil property samples was taken out to determine the soil's physical properties. We measured soil bulk density (BD), soil water content (SWC), and soil porosity (POR). After the remaining samples are spread out and wholly air‐dried, the soil samples in each plot are mixed into a composite sample to consider spatial heterogeneity within the plot. Then, the composite samples from each plot are ground into powder and screened using a sieve (with a diameter of 0.15 mm) screening of 15 g chemical soil samples to determine the soil chemical properties (SOM, soil organic matter; pH, pondus hydrogenii; TN, total nitrogen; AN, alkali‐hydrolyzable nitrogen; TP, total phosphorus; AP, available phosphorus; TK, total potassium; AK, available potassium) (Bao, 2000).

### 2.4 Data analysis

For the analyses, we first recorded the mite community in each sample site, combining abundance (ind./m^2^) and genus richness per sample unit (total number of genera per sample site). In this research, we used four diversity measures and the genus data to calculate the Shannon–Wiener diversity (*H*′) per sample unit and the Margalef richness (*D*) indices. We found many genera with lower abundance in each plot during the identification process. In order to estimate species richness and the number of unobserved species, we chose the Fisher index and ACE index (Lee, 1992). The Fisher index is a statistical diversity indicator suitable for logarithmic distribution and can effectively reflect the number of effective species in a community. The ACE index, an abundance‐based coverage estimator (ACE), considers a more comprehensive range of rare species compared to the commonly used Chao1 index and has been corrected for the coefficient of variation and sample coverage. It can be used to estimate the number of species in a community. We estimated soil mite diversity using the above indices, and the statistical software PAST 4.02 (Hammer et al., 2001) was used to calculate these indices. In order to visualize the variation of dominant genera in each ecosystem and to characterize the distribution of mites, this research used McNaughton's dominance index, which defined dominant genera, those mites with *Y* > 0.02 and those genera with ≥10% relative abundance in each ecosystem (McNaughton, 1968).

We extracted the top 10 taxa in mite abundance, categorized the remaining taxa as other, visualized the soil mite community with the sample site using Circos plots, and to further visualize unique and shared mite genera across gradients inside and outside the burrow, we used UpSetR plots, a novel visualization technique for quantitative visualization of ensemble intersections (Conway et al., 2017).

In order to explore the pattern of differences in mite composition inside and outside the cave, principal coordinates analysis (PCoA) was carried out to analyze the composition of soil mite communities under different gradients by grouping the samples in different combinations (Dixon, 2003), and testing whether the between‐group differences were greater than the within‐group differences by analysis of similarities (Anosim) test (Clarke & Warwick, 2001). Intra‐group differences were tested by Anosim to see if the differences between groups were greater than the differences within groups. Multi‐response permutation procedures (MRPP) test was used to assess the significance of the differences between the different clusters (Stallins, 2003), and all the above analyses were based on the Bray–Curtis distance matrix. Similarity percentage analysis (SIMPER) was used to identify the main taxonomic units responsible for group differences based on the Bray–Curtis dissimilarity index between communities (Clarke, 1993). To ensure that the data were normally distributed, the mite abundance and diversity indices were log‐transformed, and mite differences between the burrow's internal and external environments were analyzed using an independent samples *t*‐test, with the *t*‐test being used if the data were homogeneous, and the Mann–Whitney test is used if the data were not homogeneous (*p* < .05) (Lui & Cumberland, 2010).

Redundancy analysis (RDA) examined the relationship between Hellinger transformation mite community data and environmental variables. We first performed detrended correspondence analysis (DCA) to check the data response, which showed that the gradient length was below 4.0. Hence, RDA analysis was performed, and variance inflation factor (VIF) was applied to exclude autocorrelated environmental variables before RDA analysis. The level of significance was based on 999 Monte Carlo permutations. The direct and indirect effects of environmental variables on the response of soil mite diversity to environmental variables were investigated using partial least squares structural equation modeling (PLS‐SEM), where variables with external loadings less than 0.7 were excluded from our model. After removing some of the variables, soil organic resources were expressed as SOM, TN, and AN, and physical properties were expressed as AT, POR, SWC, and ST. The degree of convergence of the variables was assessed using average variance extracted (AVE) with composite reliability (CR), and the overall predictive power of the model was assessed using the goodness of fit (GOF) statistic.

## 3 RESULTS

### 3.1 Community composition of soil mites

**FIGURE 3:**
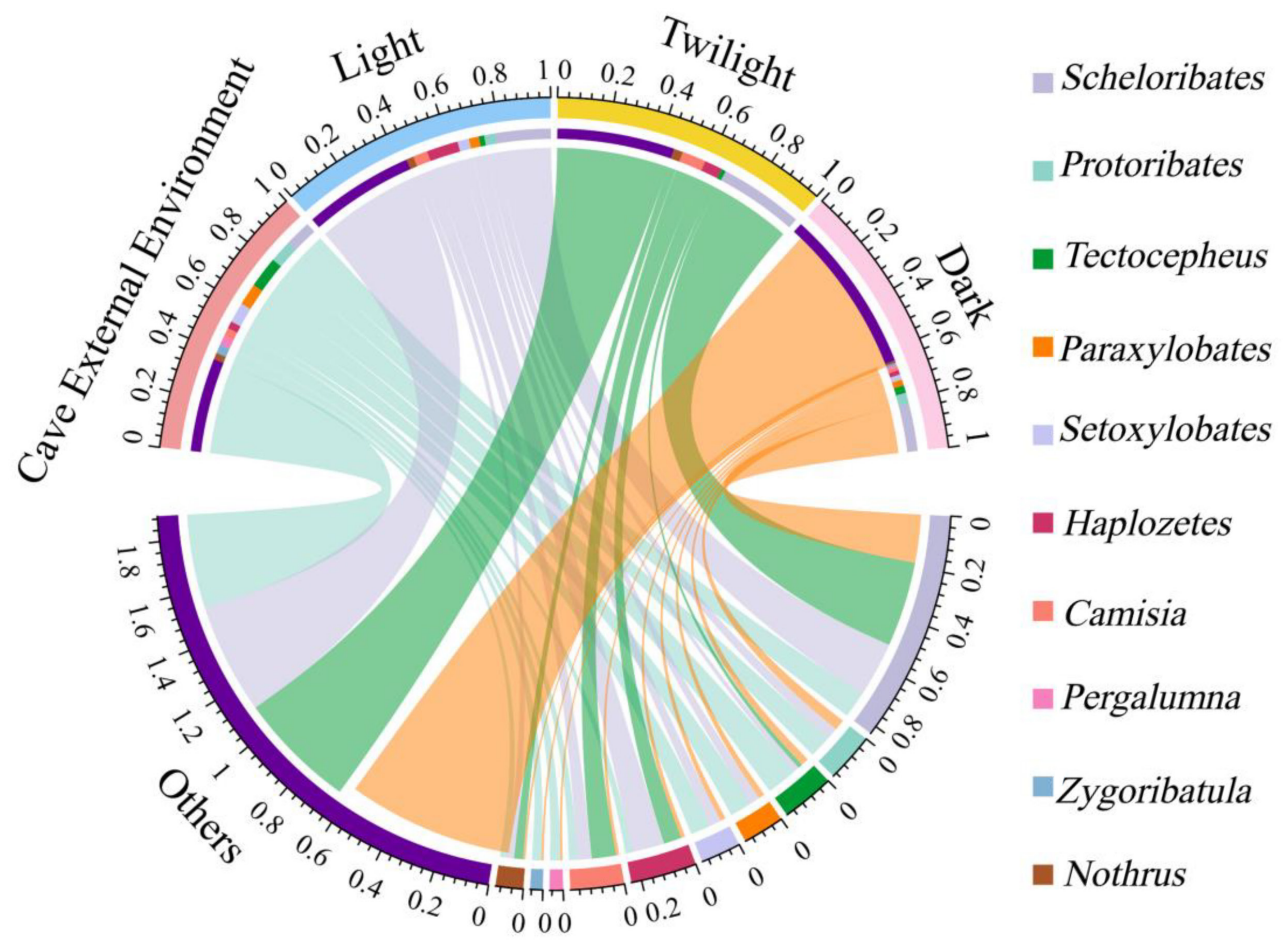
Circos diagram of the relationship between mite groups and sample plots. *Note*: Different inner color bands represent the relative abundance composition of mites. The inner color bands of different genera of mites represent their distribution in different underground environments, and the thickness of the lines represents the relative abundance of corresponding underground mite species. Only the top 10 mites with relative abundance were exhibited.

**FIGURE 4:**
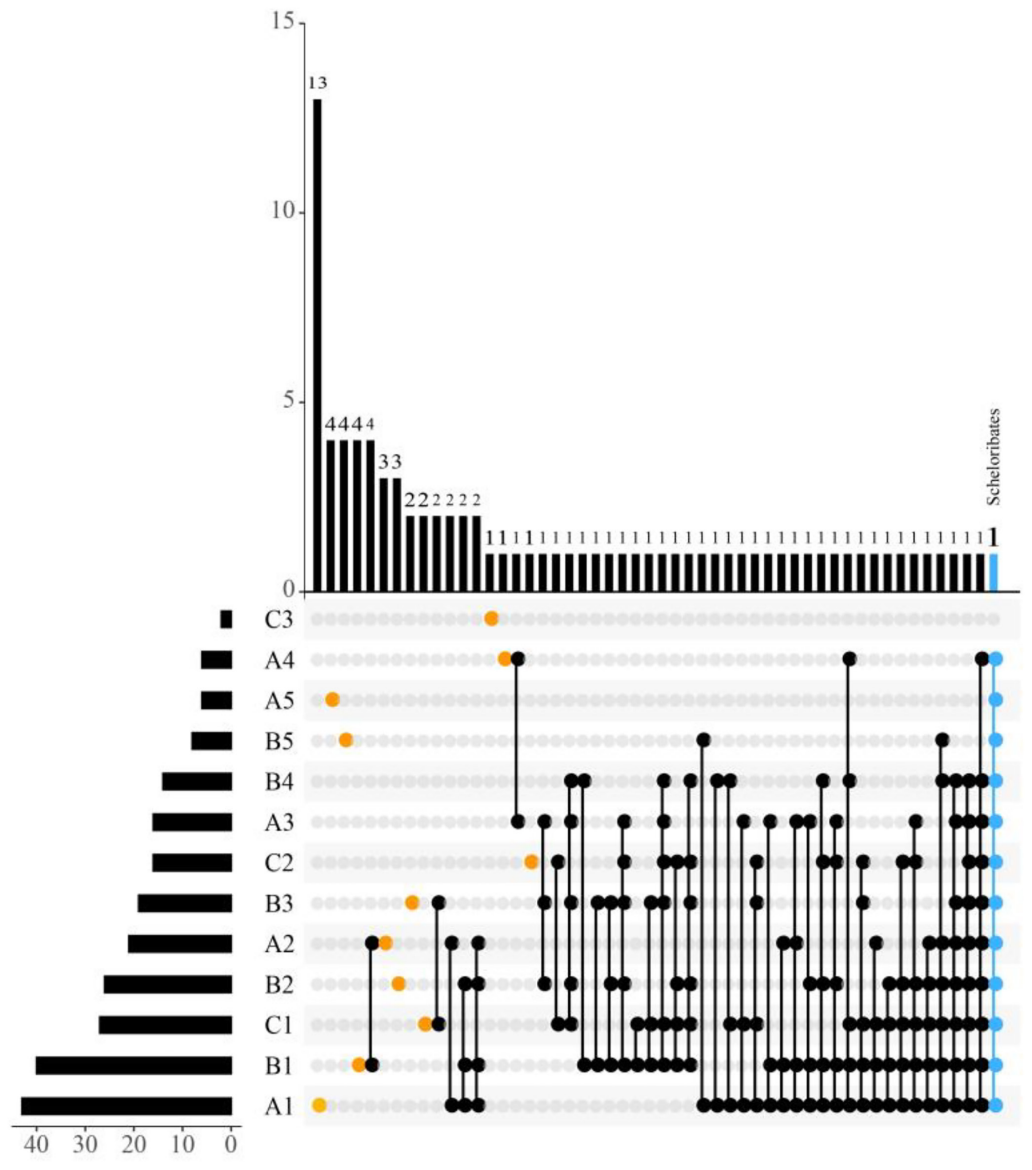
UpsetR plot of endemic and common genera under different sampling site. *Note*: The bar chart displays the unique or common genera of mites in each site, while the bar chart on the left below shows the total genera of mites in each site. The single black node represented in the figure on the right represents the number of unique mite genera in the sample site, while the black node with a connecting line represents the number of common mite genera in the statistical sample site. The endemic genus refers to mites that only appear in a location, while the common genus refers to mites that appear in at least two sites.

We identified a total of 1284 mites in 86 genera of 49 families, belonging to three main taxa: Mesostigmata (11 genera), Prostigmata (2 genera), and Oribatida (73 genera) (see list of mites in Appendix S2). Generally, *Protoribates* and *Scheloribates* were the dominant genera, accounting for 12.62% and 12.49% of the total number of individuals collected, respectively. In terms of McNaughton's dominance index, *Protoribates*, *Camisia*, *Scheloribates*, *Tectocepheus*, *Setoxylobates*, *Paraxylobates*, and *Haplozetes* were the dominant genera, accounting for 56.78% of the total number of individuals. A total of 1052 mites of 45 families and 72 genera were captured outside the cave, with *Protoribates*, *Scheloribates*, and *Tectocepheus* as dominant genera, accounting for 35.55% of the total number of mites outside the cave. A total of 232 mites of 29 families and 46 genera were captured in the interior of the cave with *Scheloribates* and *Haplozetes* was the dominant genus, accounting for 34.91% of the total number of mite individuals in the cave interior environment. The composition of soil mite communities significantly varied between the internal and external cave environments. Oribatid mites predominated in abundance within the caves (see Appendix S2). Among the top 10 relatively abundant mite species, all except *Zygoribatula* were distributed in both internal and external cave environments. Differences in the community composition of mites were observed among different environmental light zones. Six genera were distributed in the light zone, while five genera were distributed in the twilight zone, and only two genera were distributed in the dark zone. The proportion of other taxa was higher in the internal cave environment, whereas dominant taxa predominated in the external environment. Among them, Scheloribates exhibited relatively high abundance in all environmental zones (Figure 3). They were the sole mite species distributed in all habitats except for the dark zone of the Xiao cave. *Rhodacarellus* and 40 other genera were endemic to the external environment of caves, while *Cosmolaelaps, Euphthiracarus*, and 13 other genera were endemic to the internal environment of caves. Among them, three endemic taxa were found in the light zone, one in the twilight zone, and nine in the dark zone (Figure 4).

### 3.2 Community structure and diversity of soil mites

**FIGURE 5:**
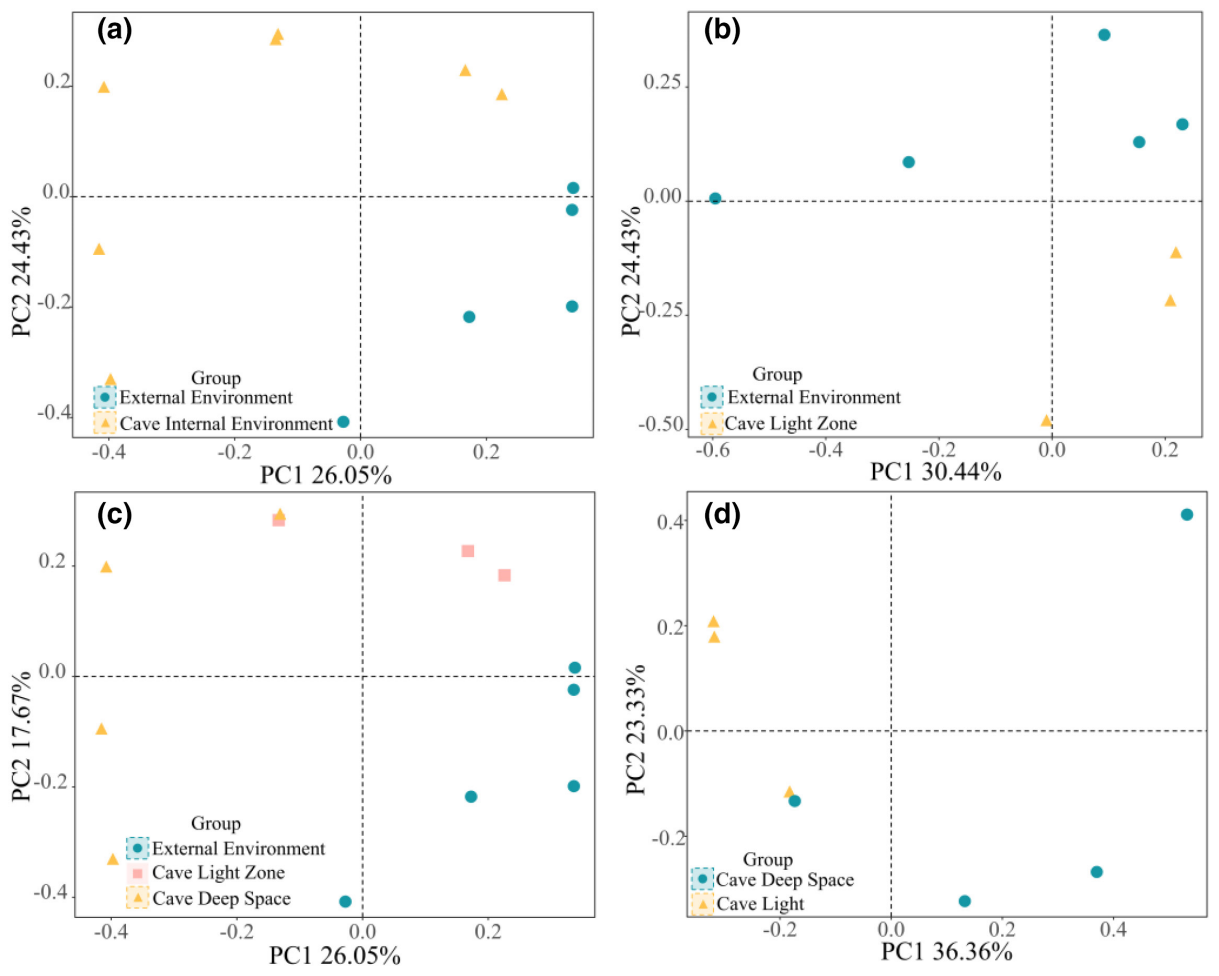
PCoA analysis of soil mite communities. *Note*: Different colored dots, triangles, and square symbols represent the distribution of abundance in different sample sites mite communities.

**FIGURE 6:**
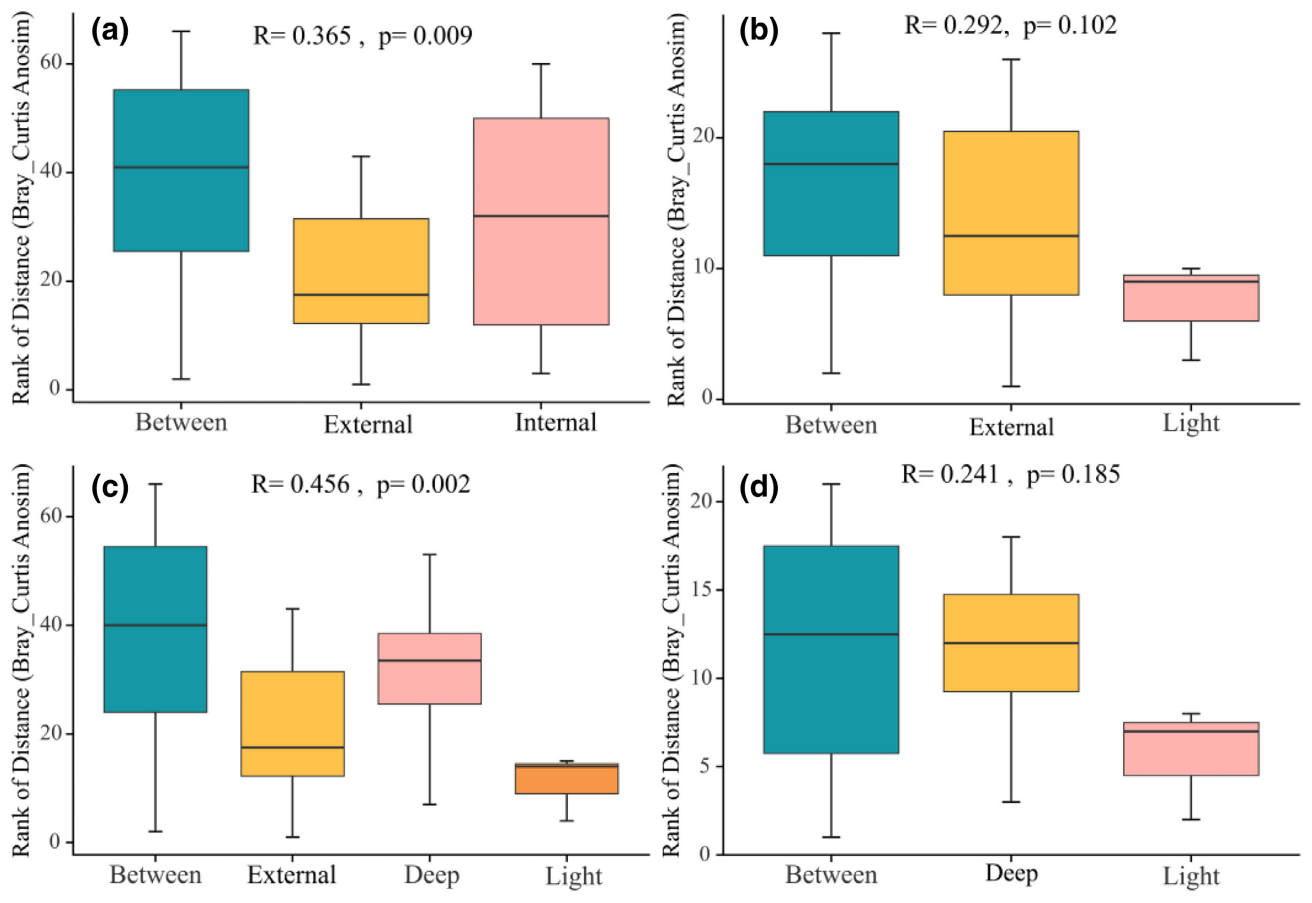
Anosim test between soil mite community groups. *Note*: The box plot is based on the Bray–Curtis distance matrix and the size indicates the score. External: Cave external space; Internal: Cave internal space; Deep: Cave deep space; Light: Cave light zone.

We first grouped the soil mite communities in the inner environment of the three caves into one group and the mite communities in the outer environment into one group. The preliminary results of the PCoA results showed that the dispersion of the two groups of samples was significant (Figure 5a). Through the Anosim analysis (Figure 6a) and the MRPP test, it can be concluded that the mite communities differed more among groups than within groups (Table 2). Furthermore, we grouped mites with light zones into one group for comparative analyses with mites from inside and outside cave environments. Comparing the outside cave environments with the cave with light zones individually, the PCoA results showed that the dispersal of the samples overlapped to some extent (Figure 5b), and the test results showed that there were significant differences in the composition of the mite communities, but that the differences between the groups were not significantly more significant than the differences within the groups (Figure 6b). Comparing the mite communities in deep cave spaces with those in the light zone, the PCoA results showed that the samples were generally dispersed (Figure 5d) and the MRPP test results showed that the differences in mite community composition were minor (Figure 6d). Overall analyses comparing the interior and exterior burrow space with the light‐banded environment showed significant differences in mite community composition (Figure 5c) with between‐group differences in the interior and exterior burrow space and the light‐banded mite community being significantly greater than within‐group differences (Figure 6c). There was some similarity in mite community composition among the three cave interior environments, but significant differences in mite composition among the different light zones.

**TABLE 2:** MRPP test for differences in soil mite community composition.

MRPP	*A*	*p*
External × Internal	0.05949	.011
External × Light	0.05518	.038
Deep × Light	0.0629	.069
Light × Deep × External	0.09503	.003

*Note*: *A*—Chance corrected within‐group agreement *A* (Corrected‐*A*), *p* < .05 indicates a significant difference.

SIMPER analysis was conducted to investigate the main mite species contributing to the differences in the communities. The results showed that *Tectocepheus*, *Protoribates*, *Paraxylobates*, *Setoxylobates*, and *Scheloribates* were the main mite species contributing to the differences in the mite communities between the external environment of the cave and the internal environment of the cave, as well as between the external environment of the cave and the lighted zone. The cumulative contribution of the differences between groups reached 45.11% and 42.65%. *Scheloribates*, *Haplozetes*, and *Parachipteria* were the major mite species contributing to the difference between the light and deep burrow environments, with a cumulative contribution of 33.95%.

Combining the results of the surveys inside and outside the cave, it was found that there were differences in the frequency of occurrence and distribution ranges of different mite species in the internal and external environments of the cave (Appendix S2). Scheloribates with the highest frequency of occurrence, accounting for 92.31% of the mites, were distributed in every habitat in the internal and external environments of the cave, with the exception of the dark zone of Xiao Cave. Camisia, Nothrus, Tectocepheus, and Microtritia appeared frequently, 76.92%, 69.23%, 61.54%, and 53.85%, respectively, of which Microtritia was also distributed in the dark zone of the caves, while the frequency of mites in the dark zone was generally 7.69%, with limited distribution, and the distribution composition of mites varied significantly among the caves. The distribution composition of mites in the dark zone of the caves varied significantly (Table 3).

**TABLE 3:** SIMPER analysis of the differential contribution of mite communities.

Zone	Ex × In	Ex × Light	In × Light	Light × Twilight	Light × Dark	Twilight × Dark	Light × Deep
	Contrib (%)						
Taxon							
*Tectocepheus*	11.96	11.67					
*Protoribates*	9.466	10.22		5.076			
*Paraxylobates*	8.37	7.933					
*Setoxylobates*	7.748	7.41		5.076			
*Scheloribates*	7.567	5.415	15.71	11.4	17.79	22.34	17.68
*Euscheloribates*						9.776	
*Neogamasus*						9.727	
*Camisia*					5.106	8.074	5.329
*Haplozetes*			11.92	12.36	11.7	6.42	10.28
*Parachipteria*			6.326	6.812	6.092		
*Achipteria*				5.126			
Cumulative %	45.111	42.648	33.956	45.85	40.688	56.337	33.289

*Note*: Contrib (%) indicates the contribution of each mite genus to the community variance and mites with a contribution greater than 5% were selected.

Abbreviations: Ex, external environment; In, internal environment.

**FIGURE 7:**
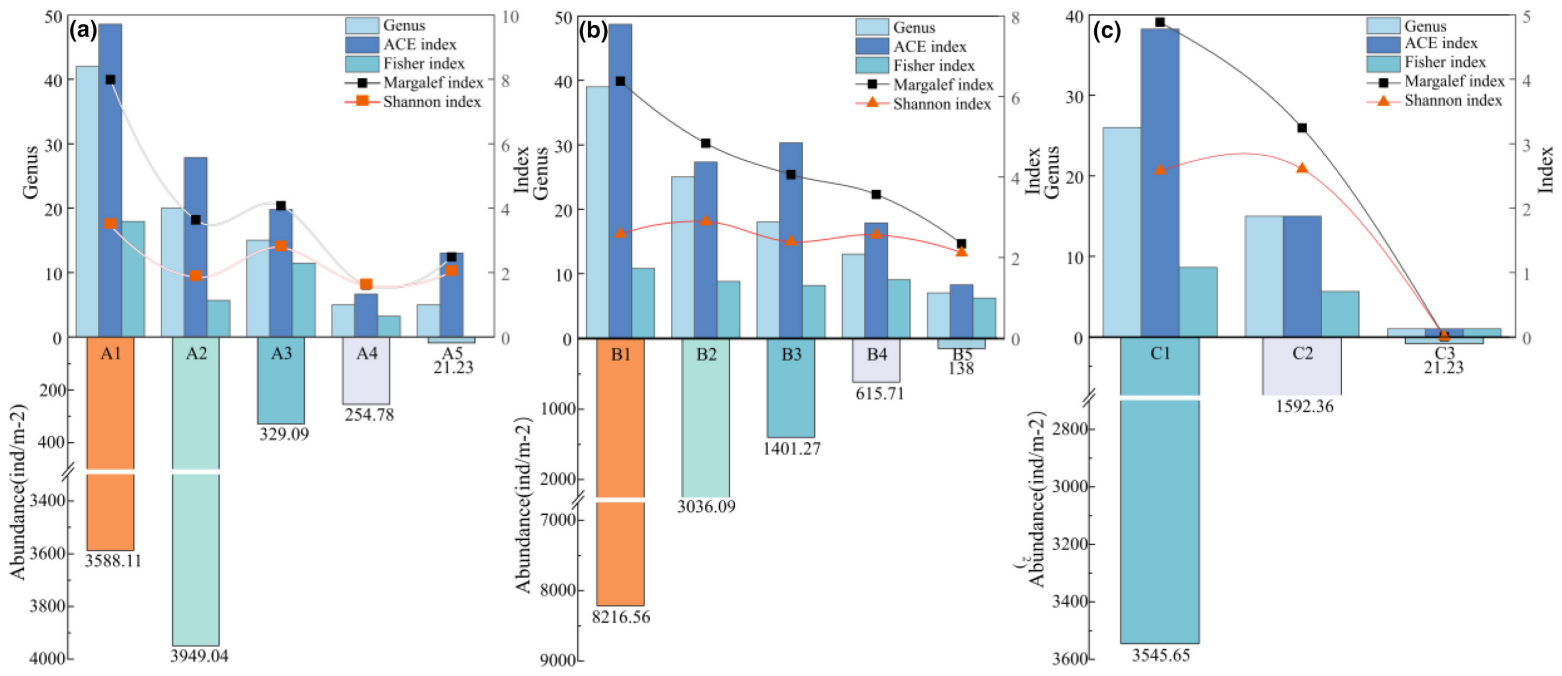
Abundance and genus richness of different cave mite communities and diversity measures. *Note*: Figures a, b, and c represent Wangtian Cave, Gan Cave, and Xiao Cave respectively. Details of the numbering in the figure are shown in Table 1.

Outside the caves, the abundance of mites ranged from 3036 to 8216 ind./m^2^, with genus richness ranging from 20 to 42 genera, and the agricultural land was relatively the least diverse habitat. Inside the caves, mites abundance and richness decreased with decreasing light intensity. Each photometric band with an overall abundance of mites ranging from 21 to 1592 ind./m^2^ and richness from 1 to 18 genera, and more significant heterogeneity in mites diversity among different cave interior environments. Wangtian Cave had a lower abundance of cave mites, ranging from 21.23 to 329.09 individuals per square meter (ind./m^2^), compared to 21.23–1592.36 ind./m^2^ in Xiao Cave and 138 to 1401.27 ind./m^2^ in Gan Cave, indicating a generally smaller population. Regarding genus richness, Gan Cave had the highest diversity (29 genera), followed by Wangtian Cave (21 genera) and Xiao Cave (16 genera). Overall, the abundance and richness of mites decreased gradually from the external to the internal cave environment. Regarding diversity parameters, the Shannon index (2.77 > 1.85), Margalef index (4.08 > 3.64), and Fisher index (11.44 > 5.69) of the mite community in the light zone of Wangtian Cave were higher than those in the external farmland. Gan Cave showed higher diversity parameters compared to the other two caves, with the ACE index predicting the richest species total of 8.27–30.31 genera, while Wangtian Cave had 13–19.8 genera and Xiao Cave had 1–15 genera. Mites in Xiao Cave were mainly concentrated in the light zone, where significant human disturbance and abundant litter resulted in fewer mites found in the deeper areas of the cave (Figure 7).

**FIGURE 8:**
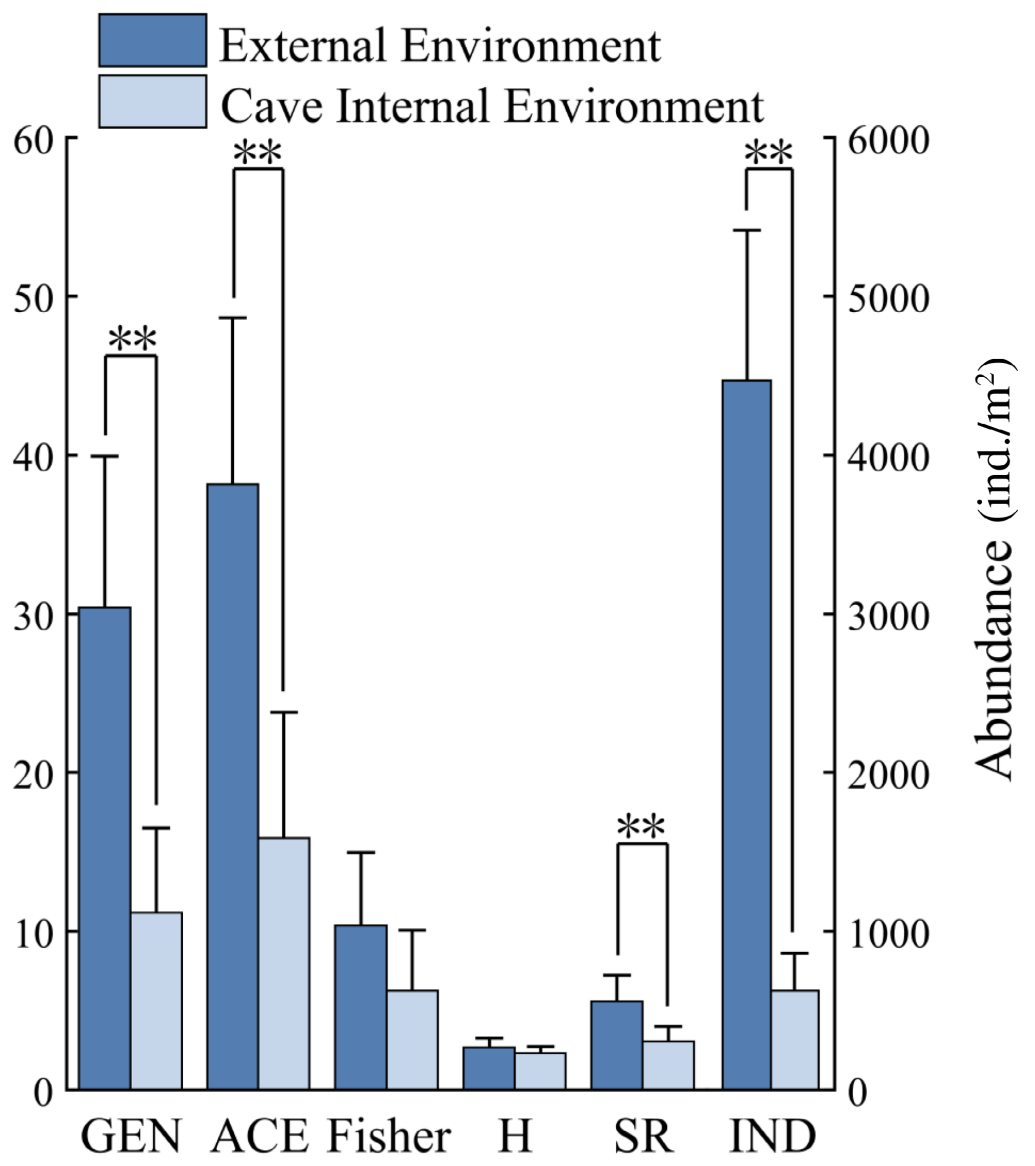
The abundance and genus richness of mite communities inside and outside caves, as well as the measurement indicators of diversity. *Note*: GEN, ACE, Fisher, H, and SR are read with left *y*‐axis values, IND is read with right *y*‐axis values, and ** indicates a highly significant difference between the inner and outer cave environments (*p* < .001). ACE, ACE index; Fisher, Fisher index; GEN, general richness; *H*, Shannon–Wiener index; IND: abundance (ind./m^2^); SR, Margalef index.

Overall, the abundance (3456.17 ± 401.96 > 600.12 ± 146.47), genus richness (26.57 ± 1.87 > 10 ± 1.61), ACE index (30.86 ± 2.47 > 14.07 ± 2.25), Fisher index (9.50 ± 0.88 > 5.83 ± 1.04), Shannon index (2.78 ± 0.12 > 2.02 ± 0.23), and Margalef index (5.07 ± 0.33 > 2.70 ± 0.36) of mites were higher in the external environment than in the cave internal environment, with significant differences in abundance, genus richness, ACE index, and Margalef index (*p* < .01) (Figure 8).

### 3.3 Response of mite communities to environmental factors

**FIGURE 9:**
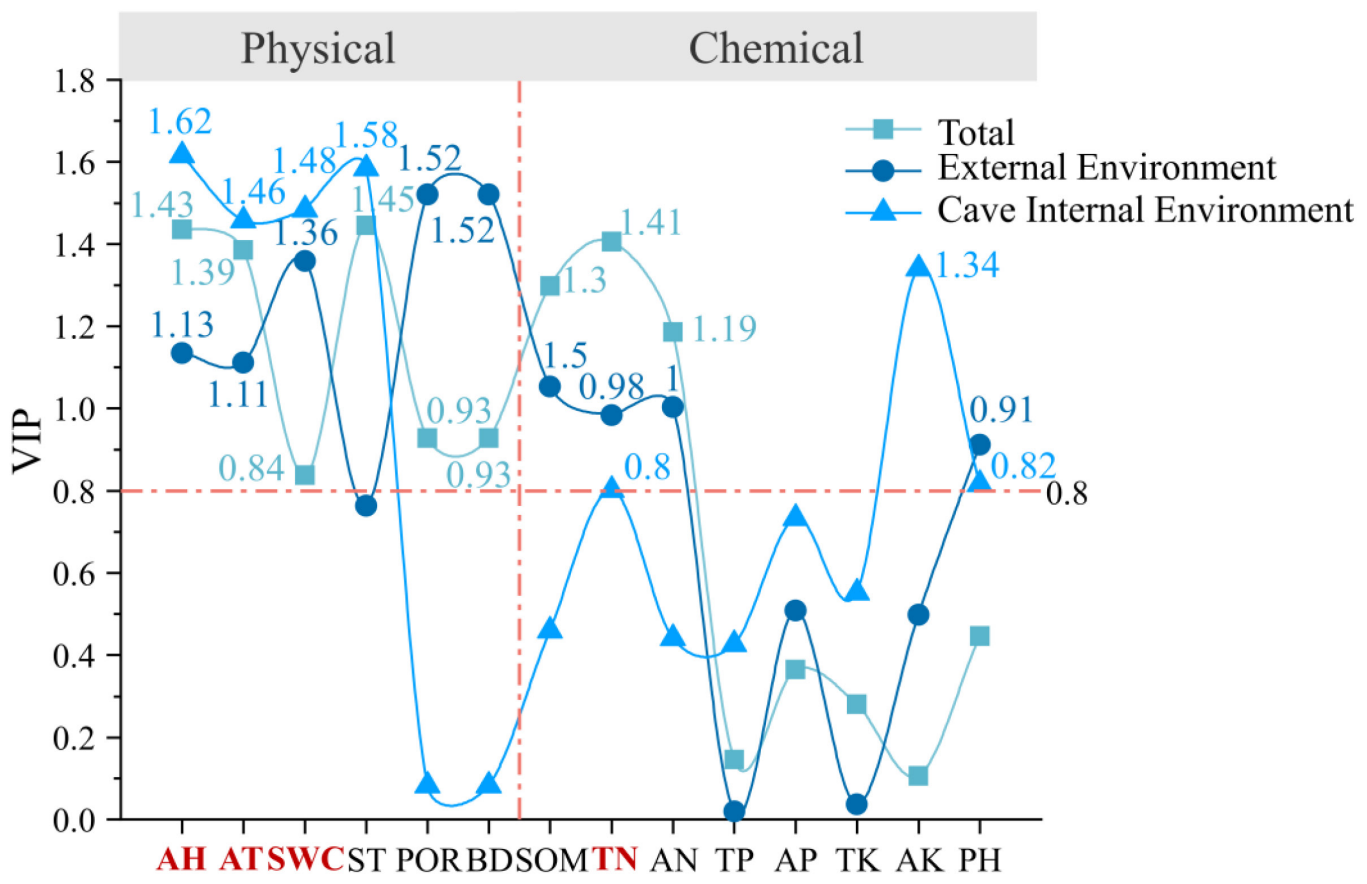
PLSR analysis of mite communities and environmental factors. *Note*: It is divided into physical environmental factors and chemical environmental factors. The environmental factors marked with numerical values are those with VIP > 0.8, while the environmental factors highlighted in red are those with VIP > 0.8 in the analysis of the overall mite community and the mite community inside and outside the cave.

The relationship between environmental factors and soil mite diversity was analyzed using the PLSR regression model (Figure 9) to investigate the critical environmental factors influencing mite diversity. Overall analysis of the relationship between the diversity of mites inside and outside the cave environment and environmental factors shows that multiple environmental factors such as ST, AH, TN, AT, SOM, AN, BD, POR, and SH have significant impacts on mite diversity (VIP > 0.8). Further, PLSR analyses of the cave's external environment showed that pH was the new significant influence factor, and ST had a relatively decreased influence. However, its VIP value was close to 0.8. In the PLSR analyses of the cave's internal environment, AH, ST, SH, AT, AK, PH, and TN were the environmental factors that had significant influences. The combined PLSR analyses showed that all four environmental factors, AH, SH, AT, and TN, were represented in the analysis of the importance of mite diversity in the internal and external environments of the cave and that the physical environmental factors had a relatively more significant influence on mite diversity in the internal and external environments of the cave.

**FIGURE 10:**
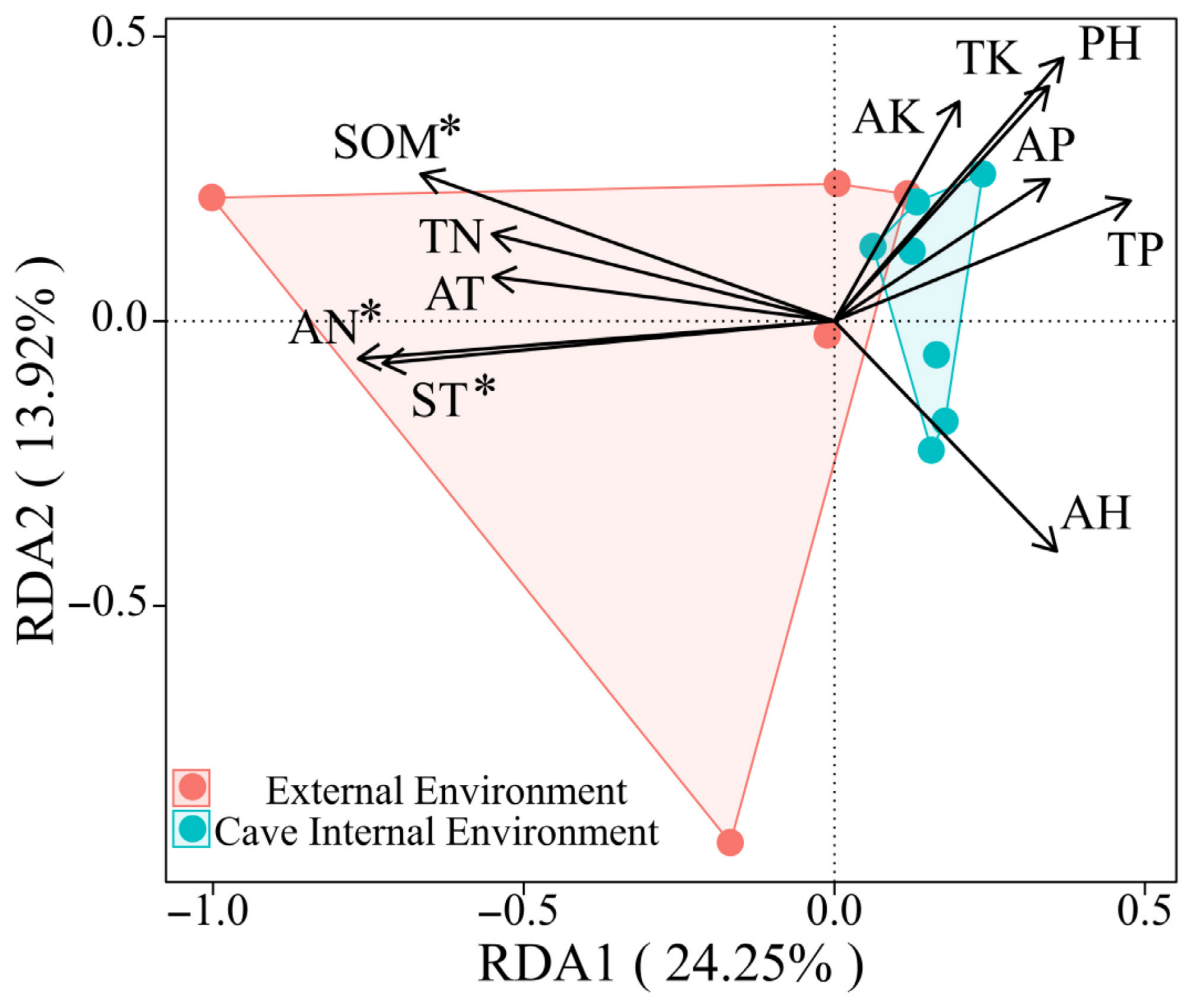
RDA analysis of mite communities and environmental factors. *Note*: * indicates significant environmental factors, *p* < .05, and the values of RDA1 and RDA2 are the percentages explained by the corresponding axes. AH, air humidity; AK, available potassium; AN, alkali‐hydrolyzable nitrogen; AP, available phosphorus; AT, air temperature; pH, pondus hydrogenii; SOM, soil organic matter; ST, soil temperature; TK, total potassium; TN, total nitrogen; TP, total phosphorus.

To investigate the interconnection of environmental and mite biological variables in the mite community in the inner and outer burrow environments, we performed redundancy analyses (Figure 10). On the first two ordination axes, RDA explained 38.17% of the variance in mite communities (axis 1, 24.25%; axis 2, 13.92%), and SOM, AN, ST, and mite communities were significantly correlated (*p* < .05).

**FIGURE 11:**
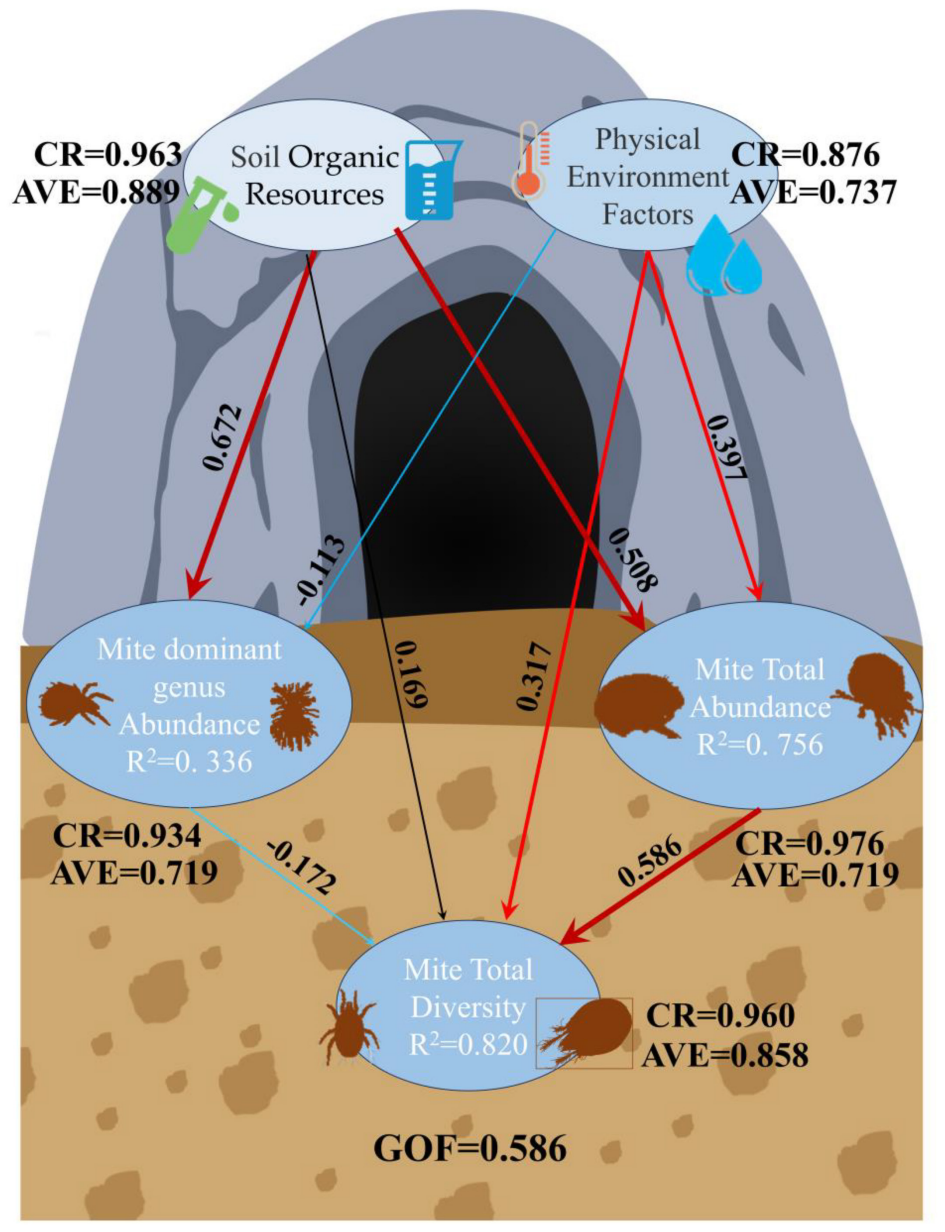
Partial least squares structural equation modeling (PLS‐SEM) shows the effect of different environmental conditions on mite abundance, dominant genus abundance, and diversity. *Note*: Numbers adjacent to arrows indicate standardized path coefficients and the width of the arrow is proportional to the strength of the association. *R* ^2^ values indicate the variance of the variables explained by the model. AVE, average variance extracted; CR, composite reliability; GOF, goodness of fit.

In the PLS‐SEM model, soil organic resources, physical environment factors, dominant genus abundance, mite abundance, and soil mite community diversity measures, five latent variables, were constructed, and their relationships were explored through path analysis (Figure 11). The results showed that soil nutrients had a strong positive effect on mite abundance (path coefficient = 0.508) and dominant genus abundance (path coefficient = 0.672) and a low effect on mite diversity (path coefficient = 0.169). Physical environmental factors had a positive effect on mite abundance (path coefficient = 397) and mite diversity (path coefficient = 0.317) and an inhibitory effect on the increase in dominant genus abundance (path coefficient = −0.113). At the same time, too high an abundance of mite dominant genera was not favorable to the growth of mite diversity (path coefficient = −0.172). The results showed significant differences in the effects of various environmental conditions on mite communities, with increased soil nutrient content promoting mite communities and temperature and humidity being critical environmental factors.

## 4 DISCUSSION

This research describes significant variations in mite community abundance and diversity in cave ecosystems in a karstic region in response to internal and external cave environments. Consistent with the central hypothesis, mite abundance, genus richness, and diversity varied with cave internal and external environments, being highest in the external environment, and mite community composition varied significantly across caves and light zones. The main taxa constituting the environmental mite community inside and outside the cave occurred between the two habitats, suggesting a specific ecological link consistent with our second hypothesis. Finally, the cave mite community responded more significantly to soil physical properties and the content of organic resources, which verified our third hypothesis. Our sample size is still insufficient due to the difficulties of sample collection for cave studies, so our generalization of these results is somewhat limited. Nonetheless, we assessed multiple variables of mite communities and environments under different caves, which allowed us to preliminarily explore associations between the biodiversity of above‐ and below‐ground ecosystems in karstic areas, and these results are generalizable for the conservation of cave ecosystems.

### 4.1 Effects of cave ecosystems on the diversity of soil mite communities

Mite abundance and richness differed significantly between internal and external cave environments, with internal cave environments being significantly lower than external environments, and there were significant differences in mite community composition between different cave environments. Mite abundance and richness decreased with decreasing light intensity in the internal cave environment, consistent with previous studies (Barczyk & Madej, 2015; Skuba et al., 2013). This result may be because caves are often considered to be simple and stable environments compared to the surface (Lunghi & Manenti, 2020; Mammola & Isaia, 2018), that environmental changes from the entrance to deeper cave locations promote gradients in environmental conditions and resources (Lunghi et al., 2014; Prous et al., 2015), and that cave interior environments can be distinctly zonal under conditions of light intensity, temperature, humidity, and availability of organic resources (Lunghi & Manenti, 2020; Peter, 2019; Tobin et al., 2013), resulting in mites with a unique composition and abundance of mite communities. In the research, we compared mite diversity in the light zone of caves with that in the inner and outer spaces of caves. The light zone of caves tends to have a small amount of vegetation distribution (Monro et al., 2018; Ren et al., 2021). Although light is still a limiting factor compared to outer space, compared to the low nutrients in the deeper spaces of the caves, the environment in the light zone provides invertebrate with a certain amount of soil nutrients, which is necessary for the survival of mites (Moseley, 2009b; Yang et al., 2021). As a transition zone connecting above‐ground and below‐ground habitats (Prous et al., 2015), species close to the light zone area may also experience daily and seasonal fluctuations in external environmental conditions (Prous et al., 2004), which also allows photic zone mites to be better adapted to their environments, allowing for higher diversity and even more stable survival compared to the outside of the cave (Lunghi et al., 2017; Manenti et al., 2015; Prous et al., 2015). On the other side, in the deep space of the cave, the stability of temperature and humidity is higher. However, the decrease in light intensity makes the organic resources scarce, and the deep space shows low temperature, high humidity, and nutrient deficiency (Lunghi et al., 2017; Moseley, 2009a). Mites surviving in deep space respond sensitively to changes in temperature and humidity, especially those in the dark zone. It is hard to find them in any other sample sites. In specific cases, these environmental characteristics observed in the subsurface environment may also be altered by disturbances caused by human activities (Ladle et al., 2012). For instance, the pollution of cave environments by rubbish dumping, which we found in Xiao Cave, and the fact that human activities can cause a rapid increase in temperature inside the cave, but it takes 6–8 times longer to return to the natural state (Calaforra et al., 2003), which in turn disrupts the stability of the mite community, as confirmed by the low abundance of mite diversity in Xiao Cave.

### 4.2 Factors affecting the composition of burrowing mite communities

Acarina was first reported to be available as troglofauna in recent years, but only for some endemic taxa (Gallao et al., 2023), and the main drivers of the pattern of distributional composition of mite communities are related to abiotic factors (temperature, humidity, and elevation), and the quality and availability of trophic resources (Laura et al., 2014). Various research has shown that the community composition of mites associated with microhabitat changes varies considerably (Wissuwa et al., 2012), and cave mites are particularly affected by environmental changes, with significant differences in community composition under different environmental conditions. The dominant genus of mites can reflect their adaptability to the environment and their sensitivity to environmental changes. *Scheloribates*, as a dominant genus common to both inside and outside cave environments in this research, is a fungivorous mite with reduced competition for food resources and better survival conditions under low‐nutrient environmental conditions compared to other mites (Sket, 1999; Zhang et al., 2023), which was found in basically all the sample plots. *Haplozetes*, the other dominant genus of mites in cave interior environments, was recorded in all interior and exterior environments. In the karst ecosystems of the region, *Scheloribates* and *Haplozetes* are also extremely important mites, acting as dominant genera in the ecological stabilization of the forests of the heritage property (Zhou et al., 2022), in the agricultural soil environment (Wei et al., 2022) and in the integrated management of rocky desertification (Chen et al., 2018) in order to indicate environmental changes. *Protoribates* were found in abundance in the Gan Cave scrub habitat. The genus has been reported as a dominant genus in heritage forests within the region (Lin et al., 2018), as well as in some extreme environments (Dou et al., 2022), where it is well adapted to environmental differences, and it is found both inside and outside of Xiao Cave, and similarly in the external environment of Xiao Cave we find a high abundance of *Setoxylobates*, which is a common mite in karst ecosystems (Chen et al., 2018; Wang et al., 2018; Zhou et al., 2022), was similarly found in the light zone, suggesting that the ecological relationship between the interior of the cave and its adjacent surface habitats is closely linked. Environmental factors, including temperature, humidity, and organic resource content, often differ significantly between caves. The relative isolation of caves limits organism dispersal, with most troglofauna confined to a single cave (Monro et al., 2018; Romero, 2009), which also significantly affects the community composition and abundance of soil mites in different caves (Monro et al., 2018; Romero, 2009). We found a higher abundance of mite communities in the deep space of Gan Cave, and the presence of many bats at the entrance and inside the cave brought a large amount of food resources for cave‐dwelling animals inside the cave (Ladle et al., 2012).

In contrast, in Wangtian Cave and Xiao Cave, only the entrance of the cave has a light zone distributed by a small amount of vegetation, where the mite community mainly gathers, which also causes a significant difference in the composition of the mite community, confirming that the diversity of the cave invertebrate is related to the distance from the entrance of the cave and the availability of the organic resources as the basis of the food. Similar studies have found the dominant family of mites to be Mochlozetidae (Yang et al., 2021) in other caves within this study. While its common taxa were found inside the caves in this study, primarily concentrated in the light zone, this study needed to be more specific to genus order, and we could not further compare the composition of the mite community. In contrast, in the European region, where the study has been carried out relatively extensively, Belgium is one of Europe's more extensive and comprehensive samplers and recorders of cave fauna. In a local study of cave mites, the common mites found in Belgium and the European region caves were recorded (Skuba et al., 2013). We did not find a similar mite composition to that in this study, possibly due to the environmental heterogeneity of the caves (Pellegrini et al., 2016; Rodolfo et al., 2018). However, species descriptions of caves depend heavily on the number of caves sampled (Culver et al., 2003), and at present, the study on cave mites in China is still very scarce.

Although caves have recently been recognized as closed or semi‐closed ecosystems (Romero, 2009), material exchange and energy flow with the external environment are highly complex (Liu, 2021). Different caves are distinct in many of their environmental attributes. However, they are relatively similar in some physical and organic resource characteristics (Souza‐Silva et al., 2021), which promote similar survival patterns for cave communities, and the high degree of environmental stability of cave interiors provides mites with similar environmental requirements and habitat characteristics. In addition to the bats found in Gan Cave, there are cave seeps and drips inside Wangtian Cave. However, most samples were collected, and we did not find more mite specimens, which may be related to the low‐temperature and high‐humidity environment inside the cave (Malica et al., 2024; Moradi et al., 2023). In this study, the physical environment had a significant effect on mite communities in our study, and cave mites are susceptible to temperature and humidity; the higher mite abundance we found in the Gan Cave may be related to the more favorable temperature and humidity conditions they possessed, with excessive humidity acting as an inhibitor to mite communities (Döker et al., 2016; Ganjisaffar & Perring, 2015). A moderate amount of organic resources promotes mite communities (George et al., 2017), and we found that AK levels were much higher in the cave's interior than in the cave's exterior, which is consistent with the findings of Yang et al. This would have a significant inhibitory effect on mite communities. Therefore, the unique ecosystem of caves makes the mite community highly selective for its habitat. In our study, the number of mite taxa endemic to the internal environment of the cave was 14, primarily distributed in the deep space of the cave, which was much lower than the 40 taxa outside the cave, which also indicated that only a tiny number of mite taxa were able to adapt to the particular environmental conditions of caves. From the outside to the deeper parts of the cave, we found that the abundance of cave‐endemic taxa gradually increased, which also indicates that there is an ecological stability zone inside and outside the cave (Souza‐Silva et al., 2021) so that mites from inside and outside the cave environment can coexist in the light zone, but in the deeper space of the cave, the mites preferred to the cave‐endemic taxa.

### 4.3 Conservation of cave ecosystems

There exist 38 subterranean biodiversity hotspots in the world, most of which are concentrated in Europe, the Americas, and Southeast Asia (Pipan et al., 2020). Due to the need for systematic cave biodiversity surveys in China, surveys of a single cave have focused on only one or a few organisms (Liu et al., 2023). In China, cave systems have yet to be recognized as a hotspot for subterranean biodiversity, which is unfortunate compared to their large numbers. Describing species is a priority for conserving cave ecology and biodiversity, as undescribed species are artificially assumed to be absent from caves and thus neglected in conservation actions (Cardoso et al., 2011). Most studies of cave organisms in China have focused on cave bats (Feijó et al., 2019) or cave fishes (Wen et al., 2022), and even some surveys of cave organisms have been incomplete (Romero, 2009), and taxonomists have shown a lack of interest in documented surveys of cave fauna, which is, of course, closely related to the difficulties of cave sampling. However, for biodiversity conservation in caves, only described species are assessed, and it is alarming that the conservation of cave ecology in China currently remains at an initiative level, with only a few regions having introduced notified responses to enhance conservation (Duan et al., 2021). In this study, although Wangtian Cave is a Tiankeng Cave with fewer human activities, many domestic wastes and extremely destructive behaviors, such as stalactite collection in the past, were found. Much waste accumulated inside Xiao Cave, and in our survey, the population density of the two areas was much higher than the environmental population carrying capacity of the karst area, and many caves with a Karst skylight cave are affected by human discarded rubbish. There are also behaviors such as agricultural activities and over‐pumping of groundwater (Monro et al., 2018), which have caused severe damage to cave ecology, especially as caves are necessary storage and sources of groundwater (Ford & Williams, 2007). Many karst areas are susceptible to the response to groundwater contamination (Sovie et al., 2022), and these destructive activities are still ongoing. Conservation of cave ecosystems, therefore, requires active action; in the past, conservation of caves may have been based more on geological, landscape, and other requirements (Silva et al., 2015), but now cave fauna is taken into account when establishing conservation units (Li et al., 2022; Moseley, 2009b; Souza‐Silva et al., 2021; Wen et al., 2022), through our investigations of the cave mite community we also aim to advocate for more systematic taxonomic and ecological studies of cave organisms, but with consideration given to adopting as far as possible a minimum sampling method and to consider the social, environmental and economic contexts for managing cave ecology.

## 5 CONCLUSIONS

By analyzing the response of cave mite communities to environmental changes, we understand the ecological relationships of mite communities in the internal and external environments of caves. This study indicates that different environmental conditions significantly affect the diversity and species composition of mite communities. In the study of ecological relationships between above‐ground and below‐ground habitats, the light zone of caves as an ecological stability zone becomes a transition zone for the survival of mite communities in both inside and outside environments of caves. The diversity of cave mite communities is related to the distance from the cave entrance and the availability of organic resources corresponding to each environmental condition. Our study shows that the environmental heterogeneity of different caves is considerable, and abiotic factors are highly correlated with the diversity of mite communities. Of course, further sampling experiments are needed to verify their relationship, and environmental factors play a decisive role in the construction of cave mite communities. However, cave ecosystems are highly susceptible to human activities, which may damage the original environmental conditions and change the relatively stable environment of the cave mite community. Overall, by documenting the diversity of cave mite communities and understanding the response of mites to different environmental conditions, our study provides basic knowledge of cave biodiversity for the conservation of cave ecosystems.

## AUTHOR CONTRIBUTIONS

**Fei Yifan:** Data curation (lead); formal analysis (lead); investigation (lead); software (lead); visualization (lead); writing – original draft (lead); writing – review and editing (lead). **Zheng Shi:** Data curation (equal); investigation (equal). **Yuanyuan Zhou:** Data curation (equal); writing – review and editing (equal). **Qiang Wei:** Data curation (equal); investigation (equal); writing – review and editing (equal). **Ying Liu:** Data curation (equal); writing – review and editing (equal). **Yan Shen:** Data curation (equal); writing – review and editing (equal). **Hu Chen:** Funding acquisition (lead); investigation (equal); project administration (lead); supervision (lead); writing – review and editing (lead).

## CONFLICT OF INTEREST STATEMENT

The authors declare no conflicts of interest.

## Supporting information


**Appendix S1**.

**Appendix S2**.

## Data Availability

Data are available in Appendix S2.
