# Supplementary material for: Distribution pattern and driving factors of mite communities in karst cave ecosystems
Source: Ecol Evol. 2024 Aug 7;14(8):e11527. doi: 10.1002/ece3.11527 (PMC11306291; doi:10.1002/ece3.11527)
Supplement: Supplementary file 1 — Appendix S1. [file ECE3-14-e11527-s002.docx]

Supplemetary information

| Cave | Zone | Family | Species |
| --- | --- | --- | --- |
| WangTian Cave | External Environment | Betulaceae | *Carpinus turczaninowii Corylus heterophylla* |
|  |  | Rosaceae | *Rubus setchuenensis ; Pyracantha fortuneana ; Rosa multiflora ; Aronia melanocarpa ; Rubus biflorus* |
|  |  | Asteraceae,Compsita | *Aster ageratoides ; Artemisia dubia ; Crassocephalum crepidioides; Bidens pilosa ; Lactuca seriola* |
|  |  | Caprifoliaceae | *Viburnum chinshanense ; Lonicera japonica ; Lonicera setifera* |
|  |  | Anacardiaceae | *Rhus chinensis Rhus punjabensis* |
|  |  | Moraceae | *Broussonetia papyrifera Morus australis* |
|  |  | Fagaceae | *Quercus serrata Quercus fabri* |
|  |  | Rubiaceae Juss | *Rubia cordifolia Paederia scandens* |
|  |  | Papilionaceae | *Vicia hirsuta* |
|  |  | Rutaceae | *Zanthoxylum armatum* |
|  |  | Lardizabalaceae | *Akebia trifoliata* |
|  |  | Fabaceae Lindl | *Millettia dielsiana* |
|  |  | Liliaceae | *Lilium brownii* var. *viridulum* |
|  |  | Verbenaceae J. St.-Hil. | *Callicarpa bodinieri* |
|  |  | Salicaceae | *Populus adenopoda* |
|  |  | Elaeagnaceae | *Elaeagnus pungens* |
|  |  | Myrsinaceae | *Myrsine africana* |
|  |  | Polygonaceae | *Fallopia multiflora* |
|  |  | Celastraceae | *Celastrus angulatus* |
|  |  | Solanaceae | *Solanum nigrum* |
|  |  | Aquifoliaceae | *Ilex* sp. |
|  |  | Ulmaceae Mirb | *Celtis sinensis* |
|  |  | Gramineae | *Miscanthus sinensis* |
| Gan Cave | Light Zone | Celastraceae | *Euonymus fortunei Celastrus angulatus* |
|  |  | Pteridaceae | *Pteris confertinervia* |
|  |  | Caryophyllaceae | *Stellaria media* |
|  |  | Buxaceae Dumort | *Buxus sinica* |
|  | External Environment | Oleaceae | *Ligustrum sinense Ligustrum lucidum* |
|  |  | Bignoniaceae | *Catalpa bungei* |
|  |  | Amaranthaceae | *Achyranthes aspera* |
|  |  | Palmae | *Trachycarpus fortunei* |
|  |  | Araliaceae | *Kalopanax septemlobus* |
|  |  | Rutaceae | *Zanthoxylum armatum* |
|  |  | Caprifoliaceae | *Viburnum chinshanense* |
|  |  | Asteraceae,Compsita | *Aster ageratoides* |
|  |  | Pteridaceae | *Pteris multifida* |
|  |  | Dryopteridaceae | *Rhizoma Cyrtomii* |

Table A1 Survey of plant species


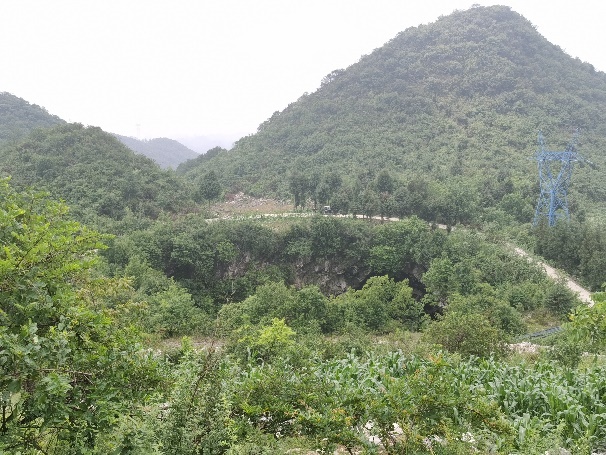

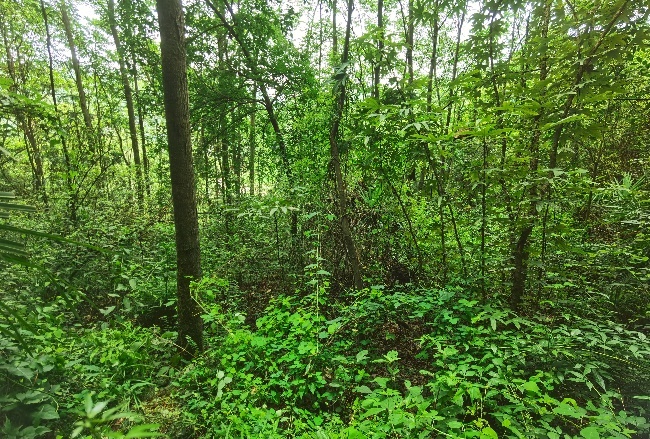


A1 B1

FigureA1 Habitat shown in our manuscript is scrub outside the cave，A1:WangTian Cave, B1:Gan Cave


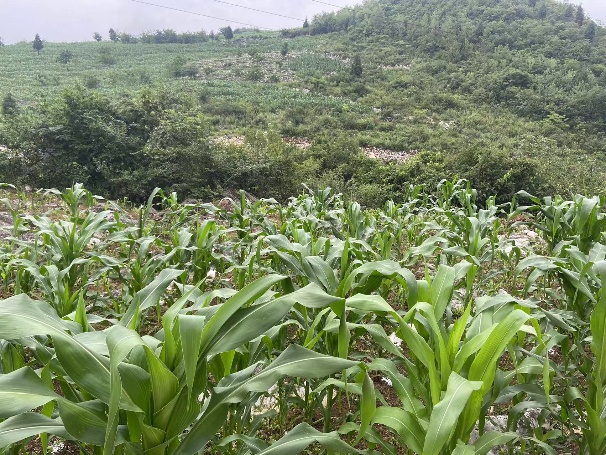

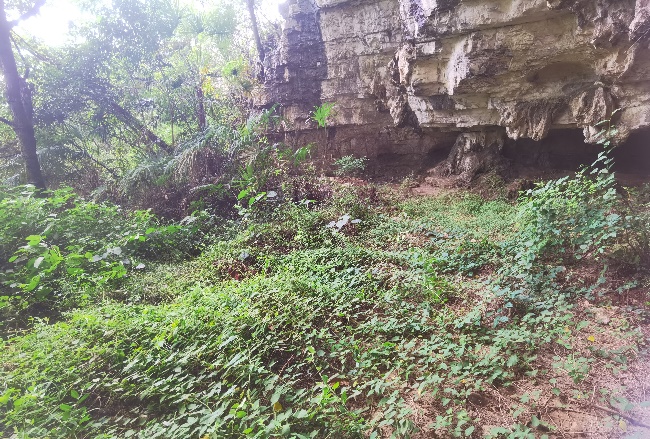


A2 B2


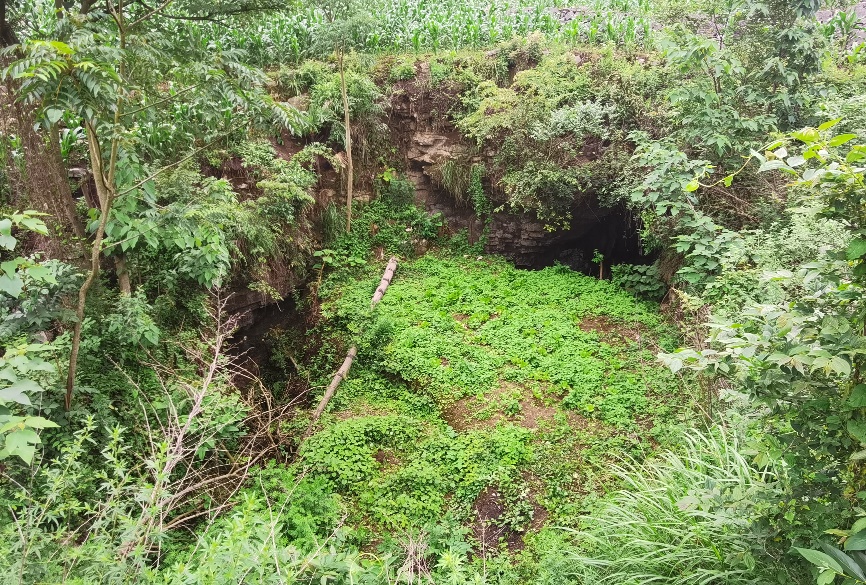


C1

FigureA2 The habitats in the figure correspond in our manuscript as，A2:WangTian Cave Corn field, B2:Gan Cave Herbaceous，C1: Xiao Cave Cabbage field


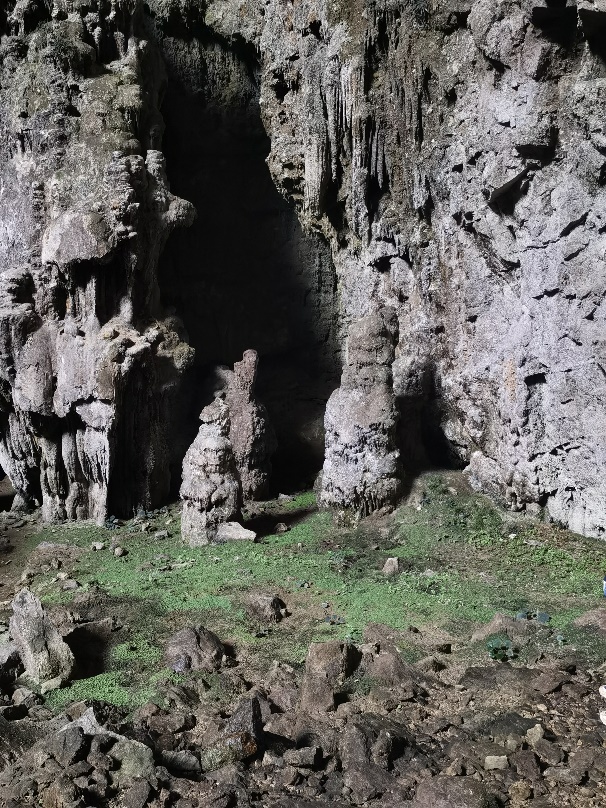

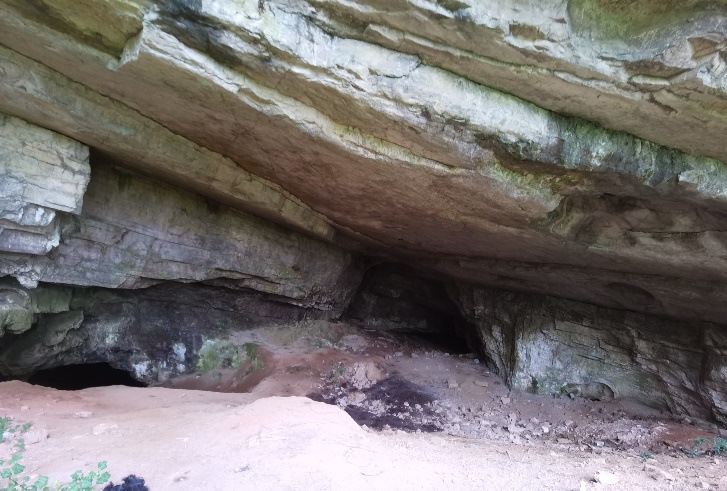


A3 B3


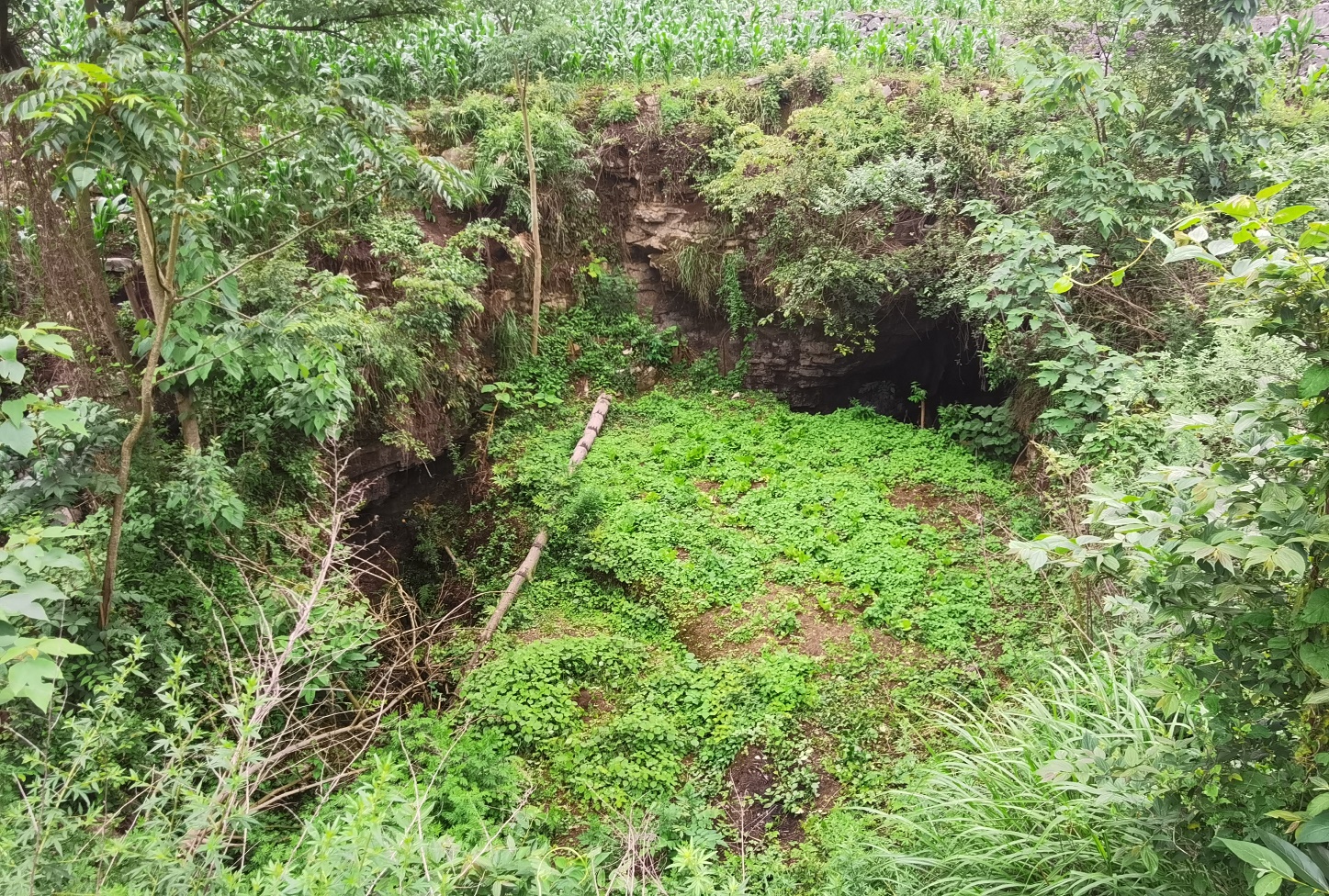


C3

FigureA3 The habitats in the figure correspond in our manuscript as，A3:WangTian Cave Light Zone,

B3:Gan Cave Light Zone，C2: Xiao Cave Light Zone


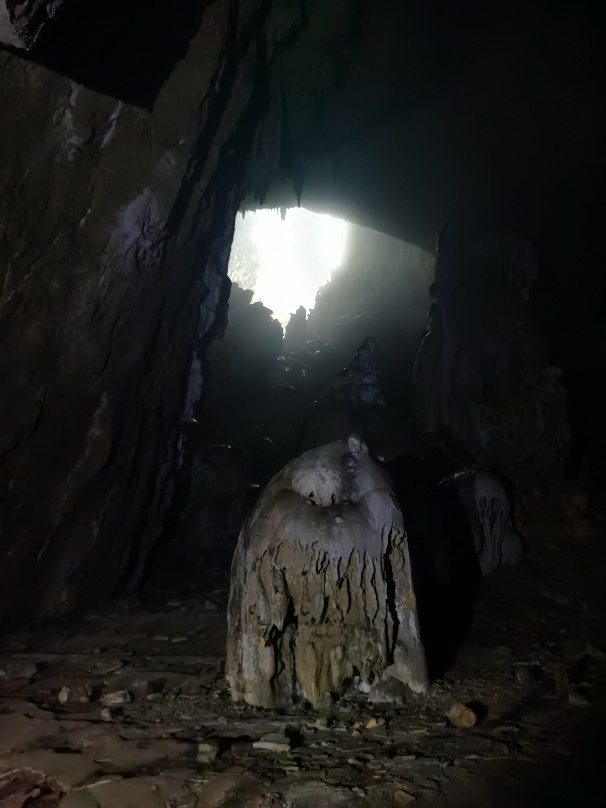

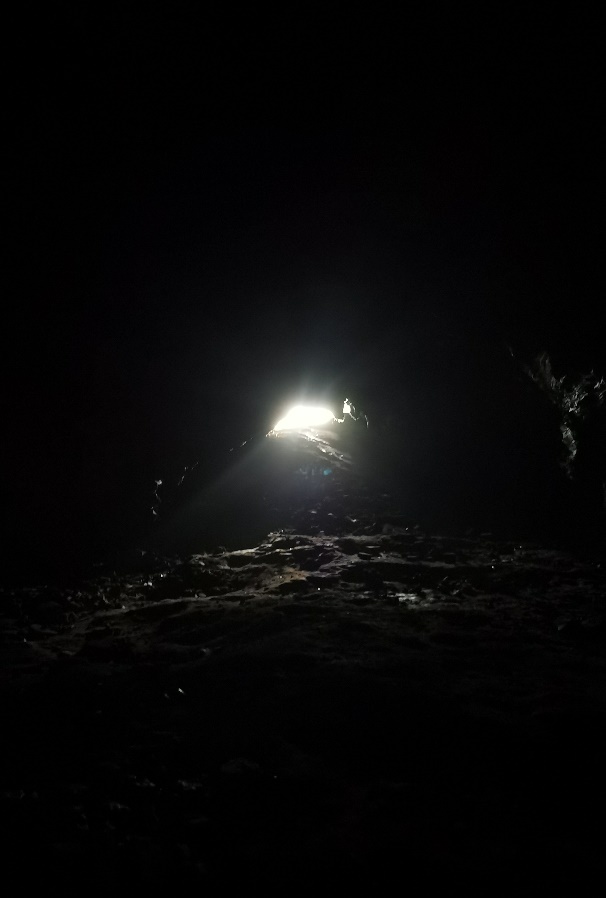


A4 B4

FigureA4 The habitats in the figure correspond in our manuscript as，A4:WangTian Cave Twilight Zone, B4:Gan Cave Twilight Zone


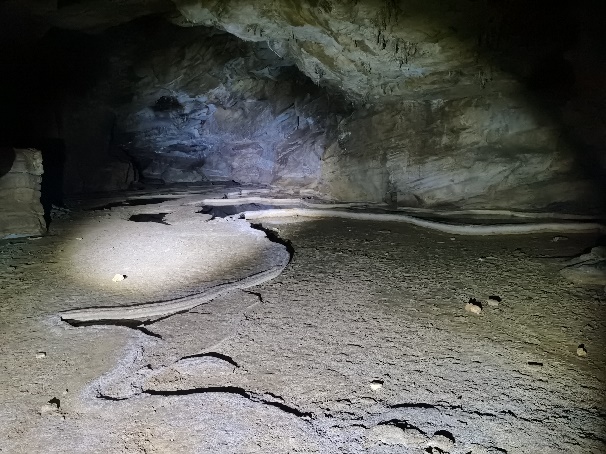

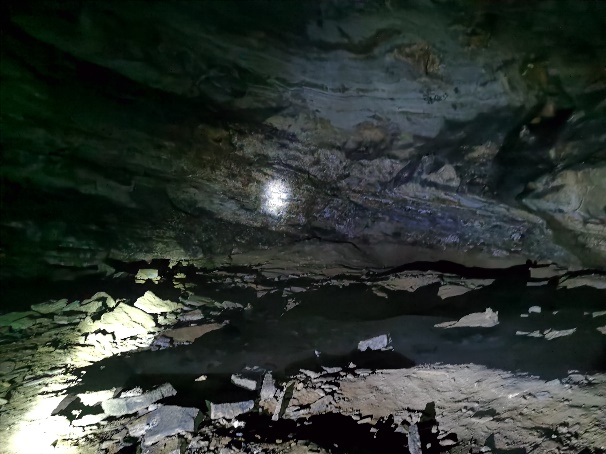


A5 B5
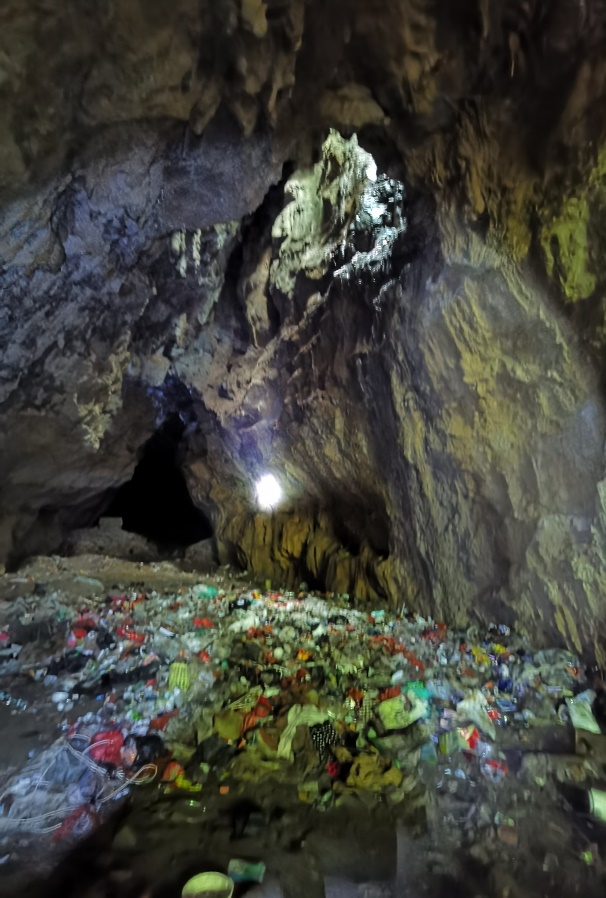

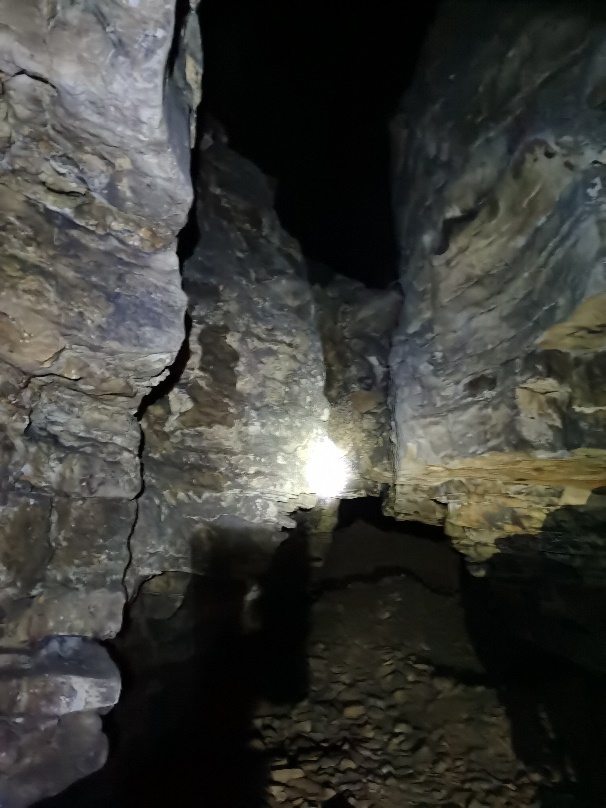


C3

FigureA5 The habitats shown in the figure correspond in our manuscript as，A5:WangTian Cave Dark Zone, B5:Gan Cave Dark Zone, C3: Xiao Cave Dark Zone
